# An improved yeast surface display platform for the screening of nanobody immune libraries

**DOI:** 10.1038/s41598-018-37212-3

**Published:** 2019-01-23

**Authors:** Tomasz Uchański, Thomas Zögg, Jie Yin, Daopeng Yuan, Alexandre Wohlkönig, Baptiste Fischer, Daniel M. Rosenbaum, Brian K. Kobilka, Els Pardon, Jan Steyaert

**Affiliations:** 1 Structural Biology Brussels, Vrije Universiteit Brussel (VUB), Brussels, Belgium; 2 VIB-VUB Center for Structural Biology, VIB, Brussels, Belgium; 3 Department of Biophysics, The University of Texas Southwestern Medical Center, Dallas, Texas 75390 USA; 4 Beijing Advanced Innovation Center for Structural Biology, Tsinghua-Peking Joint Center for Life Sciences, School of Medicine, Tsinghua University, Beijing, 100084 China; 5 Department of Molecular and Cellular Physiology, Stanford University School of Medicine, Stanford, California, 94305 USA

## Abstract

Fusions to the C-terminal end of the Aga2p mating adhesion of *Saccharomyces cerevisiae* have been used in many studies for the selection of affinity reagents by yeast display followed by flow cytometric analysis. Here we present an improved yeast display system for the screening of Nanobody immune libraries where we fused the Nanobody to the N-terminal end of Aga2p to avoid steric hindrance between the fused Nanobody and the antigen. Moreover, the display level of a cloned Nanobody on the surface of an individual yeast cell can be monitored through a covalent fluorophore that is attached in a single enzymatic step to an orthogonal acyl carrier protein (ACP). Additionally, the displayed Nanobody can be easily released from the yeast surface and immobilised on solid surfaces for rapid analysis. To prove the generic nature of this novel Nanobody discovery platform, we conveniently selected Nanobodies against three different antigens, including two membrane proteins.

## Introduction

The need for specific affinity reagents has steadily been growing to fulfil the increasing needs in diagnosis, imaging and proteomics^1^. Well-characterised affinity reagents are also instrumental for the structural and functional studies of the protein of interest. Most affinity reagents can be classified into two major subgroups: (i) antibody scaffolds and (ii) non-antibody scaffolds such as designed ankyrin repeat proteins (DARPins)^2^ or fibronectin type III like domains^3^. Nanobodies are the small (15 kDa) and stable single-domain fragments of the naturally occurring heavy chain-only antibodies, found in camelids^4^. This special IgG subclass is capable of binding to common antigenic determinants, protein flat surfaces^5,6^, peptides^7^ and small haptens^8,9^ comparably to conventional antibodies. Minor structural differences in the heavy-chain antigen binding domain, including longer complementarity-determining regions (CDRs)^10^ make many Nanobodies able to adopt a prolate shape to bind into cavities^11^. Indeed, several Nanobodies have been shown to bind deep protein cavities including enzyme active-sites^12^, and G-protein binding cavities on G protein-coupled receptors (GPCRs)^13–16^.

Several *in vitro* display methods have been developed that allow the efficient selection of affinity reagents from large molecular libraries in small volumes^17,18^. Nanobodies have been successfully recovered from immune or non-immune libraries using phage display^12,19^ and ribosome display^20^ in combination with panning. More recently, target-specific Nanobodies have also been selected by bacterial^21^ or yeast^14,22,23^ surface display followed by cell sorting. The major advantage of cell-surface display is the compatibility of these methods with the quantitative and multi-parameter analysis offered by flow cytometry^24^. In this connection, each individual cell of the library can be investigated one by one for the display level of the cloned affinity reagent and its antigen occupancy in real time^18^, under well-controlled conditions including buffer composition, pH, temperature and antigen concentration. Accordingly, high-throughput fluorescence-activated cell sorting (FACS) allows the selection and recovery of separate cell populations, displaying binders with different predesignated properties.

**Figure 1:**
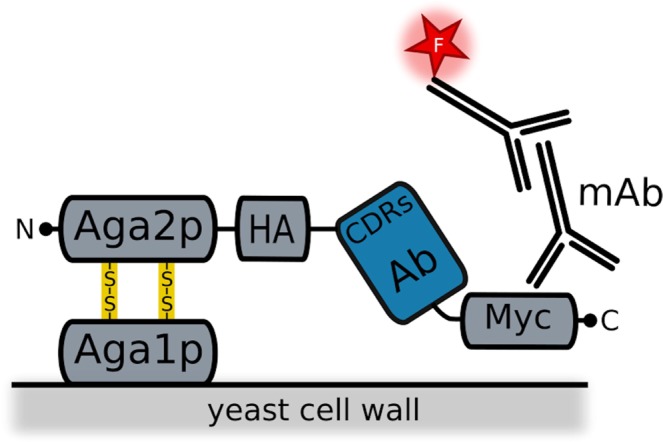
Conventional yeast surface display system for the screening of antigen-binding scaffold libraries (Adapted from^48^). Affinity reagents, including single-domain antibodies (blue) can be fused via its N-terminal end to the C-terminus of Aga2p. Surface expression can be detected by using fluorescently labelled antibodies that bind the Myc or HA tags.

*Saccharomyces cerevisiae* cells, displaying up to hundred thousand copies of a unique affinity reagent fused to the N-terminal end of the Aga2p subunit^18^ (Fig. 1) are now widely used as an alternative for display methods based on filamentous phage. For the discovery of Nanobodies, we aimed at improving this standard system in two key aspects. Firstly, the N-termini of the heavy and light chain variable domains of all subtypes of immunoglobulins are in proximity to the CDRs, responsible for antigen recognition^25^. As described previously for scFv antibody scaffold, fusions to the N-terminus of these binding domains may interfere with antigen binding^26^. A second aspect of the current systems relates to the complexity of the process to analyse the display level of the cloned immunoglobulin on the surface of the yeast cell. Most vectors cause the protein of interest to be displayed as a fusion with a peptide tag. Surface display is then quantified by use of a tag-specific primary antibody, often followed by incubation with a fluorophore-conjugated secondary antibody^18,27–32^. Considering possible reproducibility issues with commercial antibodies^33,34^ and batch-to-batch differences of antibody/fluorophore labelling ratios, consistency from experiment to experiment can be a challenge.

**Figure 2:**
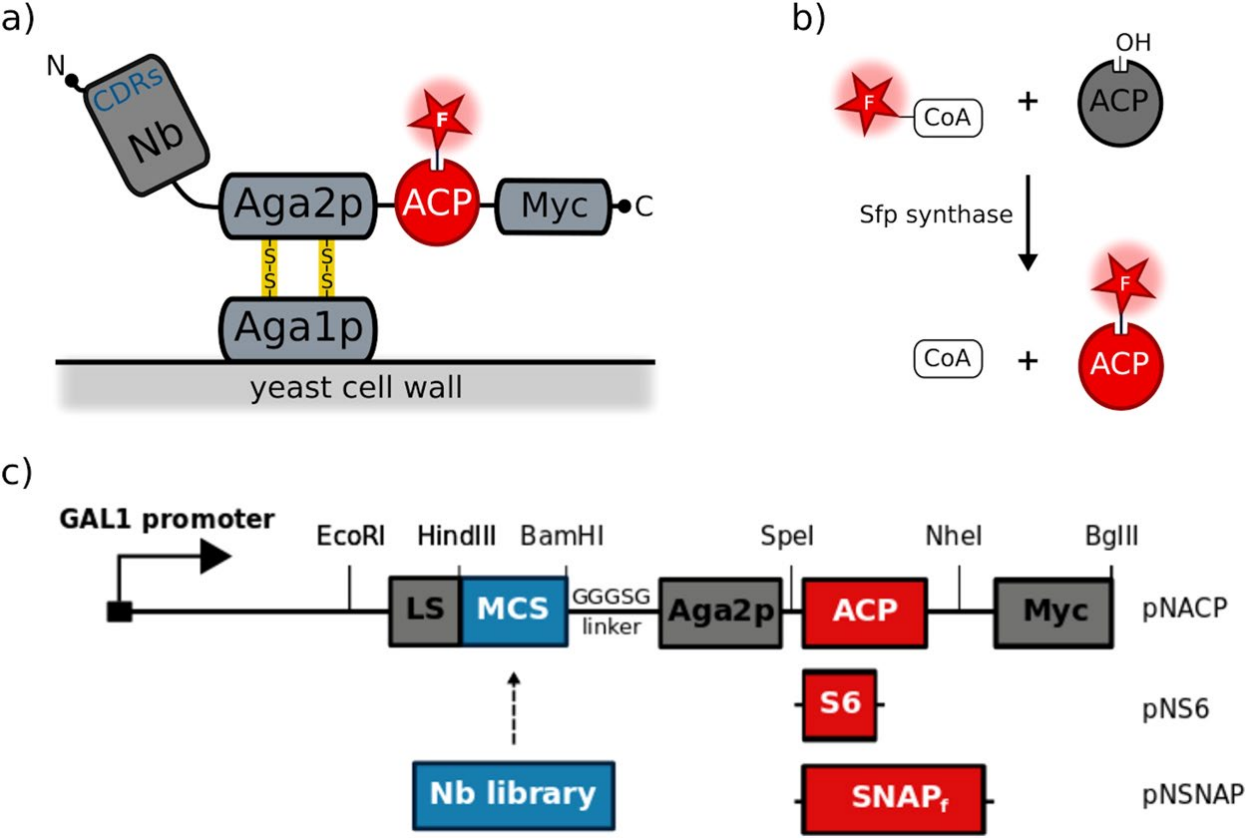
Optimised yeast display system for the screening of Nanobody libraries by cell sorting. (**a**) We designed novel vectors for the extracellular display of fusion proteins consisting of a Nanobody followed by Aga2p and by ACP. The Nanobody is fused at its C-terminus to Aga2p, leaving the CDRs fully exposed for antigen binding. (**b**) Enzymes such as Sfp Synthase can be used to covalently attach CoA derivatives containing fluorophores or biotin to a unique serine residue of the C-terminal ACP tag. (**c**) In our vectors, Nanobody libraries can be cloned as fusion proteins under the transcriptional control of the GAL1 promoter. The fusion protein is secreted by using the appS4 leader sequence. ACP can be replaced by other tags that are amenable to covalent orthogonal labelling of the displayed fusion protein: S6 (pNS6 vector) or SNAPf (pNSNAP vector).

In order to overcome these hurdles, we present an alternative yeast display system where each Nanobody is fused at its C-terminus to the N-terminus of Aga2p (Fig. 2a). Moreover, the display level of a cloned Nanobody on the surface of an individual yeast cell can be monitored through a covalent fluorophore that is attached in a single enzymatic step to an orthogonal acyl carrier protein (ACP) tag^35^. To prove the generic nature of this novel Nanobody discovery platform, we conveniently selected Nanobodies against human OX_2_ orexin receptor, human α_2A_ adrenergic receptor and human coagulation Factor IX.

## Results and Discussion

### Design of a novel Nanobody surface display vector for yeast

Exploiting the exquisite display properties of the yeast a-agglutinin system^18^, we designed a novel pNACP vector system allowing the display of fusion proteins containing a Nanobody followed by Aga2p and by ACP (Fig. 2a). ACP corresponds to the small 78 amino-acid long acyl carrier protein of *E*. *coli*^36^ that is used as a versatile tag. Coenzyme A (CoA) derivatives, including fluorescent or biotinylated variants can be enzymatically coupled through a covalent bond to a conserved serine residue by the phosphopantetheinyl transferases from *Escherichia coli* (AcpS) or *Bacillus subtilis* (Sfp) (Fig. 2b). Utilising the *in vivo* homologous recombination machinery of yeast^37^, diverse *in vivo* matured Nanobody libraries can be easily cloned into this yeast display vector. Induction of the GAL1 promoter triggers the display of the Nanobody repertoire on the surface of the yeast cells. A stop codon was introduced into the multiple cloning site (MCS) of the pNACP cloning vector in order to avoid the display of fusions that do not contain an antibody (Supplementary Fig. 1). Two analogous vectors encoding the S6 tag^38^ or the SNAP_f_ tag^39^ instead of the acyl carrier protein were also generated as an alternative to the ACP tag for monitoring the Nanobody display level (Fig. 2c). As confirmed by microscopy and flow cytometry (Supplementary Fig. 1), all three vectors enabled the multivalent display of Nanobodies on the surface of yeast but ACP fusions consistently showed the highest measurable display levels. Accordingly, we focused on ACP fusions to further validate our Nanobody yeast display system.

### One pot, one step reaction for the orthogonal labelling of displayed Nanobodies on the surface of yeast

**Figure 3:**
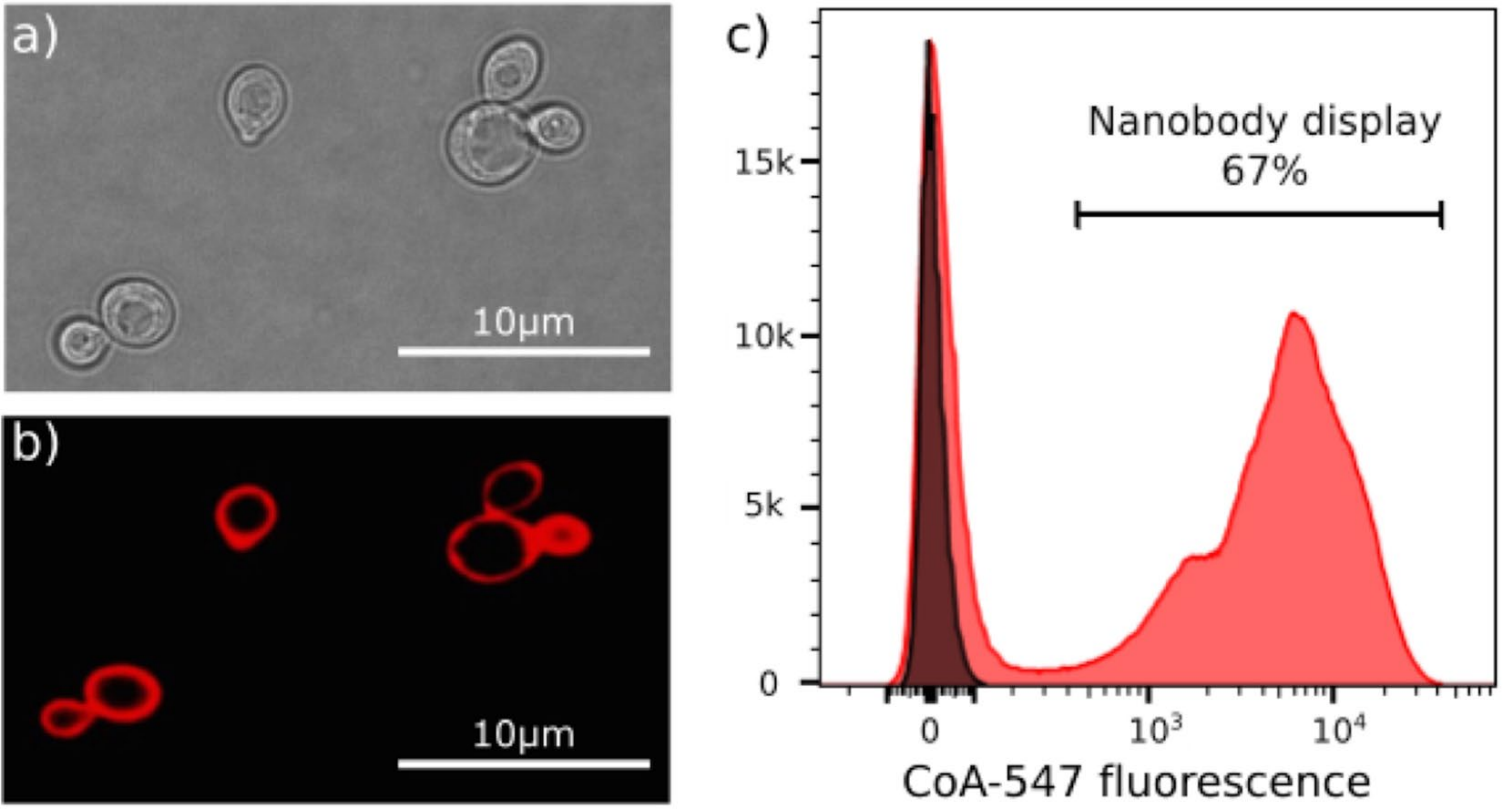
Orthogonal labelling of displayed Nanobodies on the surface of yeast. EBY100 yeast cells containing pNACP_Nb35 were grown and induced overnight in galactose-rich medium. The Nb35-Aga2p-ACP fusion was orthogonally labelled by incubating these cells with CoA-547 in the presence of Sfp synthase. Surface display of the fusion protein was analysed by light microscopy (**a**) and confocal microscopy (**b**,**c**) Histogram of the flow cytometric analysis of CoA-547 labelled cells expressing the fusion (pNACP_Nb35, red), compared to labelled yeast cells that do not display a Nb-Aga2p-ACP fusion (pNACP, grey). The fluorescence intensity of each cell was monitored at 582 nm upon excitation with a 561 nm yellow-green laser.

Current systems fuse the protein of interest to peptide tags such as Myc tag to monitor the surface display on each yeast cell by reversible labelling with tag-specific antibodies in combination with fluorophore-conjugated secondary antibodies. Here we present Nanobody-Aga2p-ACP fusions that are secreted from yeast by the appS4 leader sequence^40^ to covalently associate with Aga1p on the surface of the cell^18^. With this system, the extracellular display is monitored robustly by labelling the fusion covalently with a fluorescent dye in a one pot, one step enzymatic reaction (Fig. 2b). To validate this new orthogonal labelling technique, we cloned a well-characterised Nanobody (Nb35) that binds the μ-opioid receptor^15^ into pNACP. EBY100 yeast cells bearing this plasmid (pNACP_Nb35) were grown and induced overnight in a galactose-rich medium to trigger the expression and secretion of the fusion. For the orthogonal staining of ACP, cells were next incubated for 1 h in the presence of catalytic amounts of the Sfp synthase and 2 μM fluorescently labelled CoA analogue (CoA-547) as a substrate to covalently attach the DY-547 fluorophore. Confocal microscopy of the stained yeast cells confirms the homogenous fluorescent labelling of the yeast surface (Fig. 3), indicating that the Nb35-Aga2p-ACP associates attachment to the yeast cell wall through disulphide bonds to Aga1p^41^. Because Sfp is a recombinantly purified enzyme that does not enter the cells, flow cytometry analysis/sorting can be used for the high-resolution separation of Nanobody displaying from non-displaying yeast cells (Fig. 3c). We also analyzed a cell-wall protein fraction by western blotting (Supplementary Fig. 2) to confirm that the displayed Nb35-Aga2p-ACP fusion is not degraded. Moreover, orthogonally stained yeast cells can be stored in the dark at 4 °C for one week with minor losses of the staining levels (Supplementary Fig. 2). Other fluorescent or non-fluorescent CoA derivatives can be easily synthesised from commercially available building block following a straightforward protocol^35^ (Supplementary Fig. 3).

### Yeast surface display for the screening of *in vivo* matured Nanobody libraries

After confirming the efficient display and fluorescent labelling of a well characterised Nanobody, we tested this new system for the selection of Nanobodies from immune libraries by yeast display followed by FACS. Three different antigens were chosen, from easy-to-target soluble proteins to difficult-to-target membrane proteins: (i) coagulation factor IX (FIX) representing a soluble protein and (ii) human α_2A_ adrenergic receptor (α_2A_AR) and human OX_2_ orexin receptor (OX_2_R) representing two GPCRs. Membrane proteins often suffer from low stability, especially after removing them from their native membrane environment. Another challenge for selecting antibodies against membrane proteins relates to the small surfaces that they expose from the lipid-bilayer. Yeast display, combined with selection by FACS requires fluorescently labelled antigen. To test the versatility of this platform, each of the chosen antigens was differently labelled: FIX was labelled covalently at its active site serine with a fluorescent suicide inhibitor, OX_2_R was labelled using a fluorescent mAb whereas α_2A_AR was tagged with a *N*-hydroxysuccinimide reactive fluorophore.

Three different llamas were immunised separately with each target following standard procedures^19^ to generate robust immune responses against these antigens (Supplementary Fig. 4). After six injections, blood samples were collected to prepare immune libraries. Accordingly, the open reading frames encoding Nanobodies were amplified by RT-PCR. PCR products were mixed with linearised pNACP and electroporated into EBY100 yeast cells (details in Methods) to generate three immune libraries by *in vivo* homologous recombination against FIX (Lib191, 1.6 × 10^7^ individual transformants), α_2A_AR (Lib192, 2.5 × 10^7^ transformants) and OX_2_R (Lib197, 3.5 × 10^7^ transformants), respectively.

### Selection of Nanobodies against activated human coagulation Factor IX

Coagulation Factor IX (FIX) plays a crucial role in the blood coagulation cascade. This protease is produced and secreted as an inactive zymogen. Physiological activation of FIX involves proteolytic cleavage to produce a two-chain form linked by a disulphide bridge, known as FIXa^42^. In this study, we used a truncated variant of FIX^43^. For the immunisation, recombinant FIX was activated *in vitro* with human Factor XIa (Coachrom) and further stabilised in its active state transition state conformation^44^ by covalent inhibition with the peptidic suicide inhibitor PPACK (_D_-Phenylalanyl-_L_-prolyl-_L_-arginyl chloromethyl ketone) that binds covalently to the active site Ser195 and His57 of the catalytic triad. A fluorescein isothiocyanate (FITC) conjugate of the same suicide inhibitor was used to label FIXa covalently for cell sorting (FIXa-FITC).

**Figure 4:**
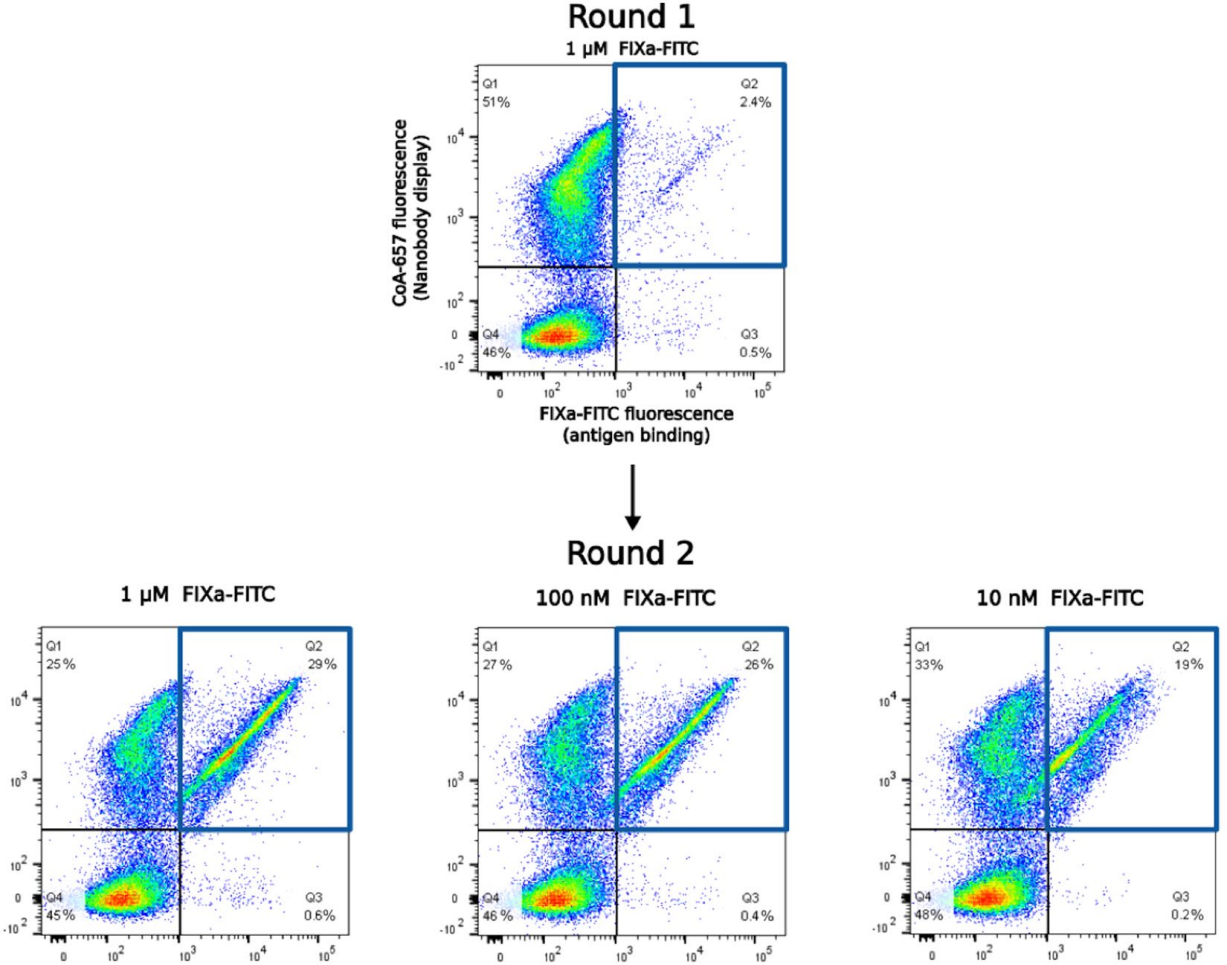
Consecutive rounds of selection of Nanobodies that bind FIXa by yeast display and two-dimensional flow cytometry. Each dot represents two fluorescent signals of a separate yeast cell of the (sub) library. The x-axis is a measure of the amount of the fluorescent antigen that is bound (FITC fluorescence) whereas the y-axis gives an indication of the Nanobody display level (DY-647P1 fluorescence). In this example, the yeast cells displaying FIXa FITC specific Nanobodies (gate Q2 represented as a blue square), were enriched from 2.4% in the first round of selection to 19% in the second round at the lowest antigen concentration (10 nM of FIXa-FITC).

Yeast cells containing display library 191 (Lib191) were grown and induced overnight. To monitor the Nanobody display density on each individual clone, 4 × 10^7^ cells were orthogonally stained with Sfp synthase using CoA-647 as a substrate to covalently attach the DY-647P1 fluorophore (details in Methods). To measure antigen binding, these yeast cells were incubated in a small volume (500 μl) with FIXa-FITC (1 μM). Finally, this double stained library was analysed by FACS. Each individual yeast cell was examined simultaneously for its Nanobody display level (fluorescence of the CoA-647 fluorophore) and antigen binding (fluorescence of the FITC fluorophore) by two-dimensional flow cytometric analysis (Fig. 4). Yeast cells scoring above a threshold fluorescence in both channels were collected (Q2 gate, Fig. 4), grown and used as the input for a next round of selection. In the second round FIXa-FITC concentrations was reduced to 1 μM, 100 nM or 10 nM, respectively (Fig. 4).

After two rounds of selection, collected cells were plated and 96 individual yeast colonies from each selection condition were picked for further characterisation. Accordingly, cells were grown overnight in 96 deep-well plate format. From each culture, one aliquot was stored at −80 °C, another fraction was used to determine the sequence of the encoded Nanobody (details in Methods) and the remaining fraction was induced overnight to analyse the binding properties of the displayed Nanobody. 10^6^ induced cells were stained orthogonally with Sfp and CoA-647. 10^5^ cells thereof were incubated with 100 nM of the FIXa-FITC fluorescent antigen. Samples were then applied on a FACS Fortessa analyser for the high-throughput identification of Nanobodies with affinities in the nanomolar range. For each individual clone, the FITC Mean Fluorescence Intensity (MFI) was compared to a negative control (Supplementary Fig. 6). Following this straightforward procedure, thirteen different Nanobody sequence families, capable of binding to FIXa-FITC, were identified (Supplementary Table 1). Nanobodies from the same sequence family derive from the same B-cell lineage and bind the same epitope^19^.

### Assessing Nanobody binding affinities by flow cytometry

**Figure 5:**
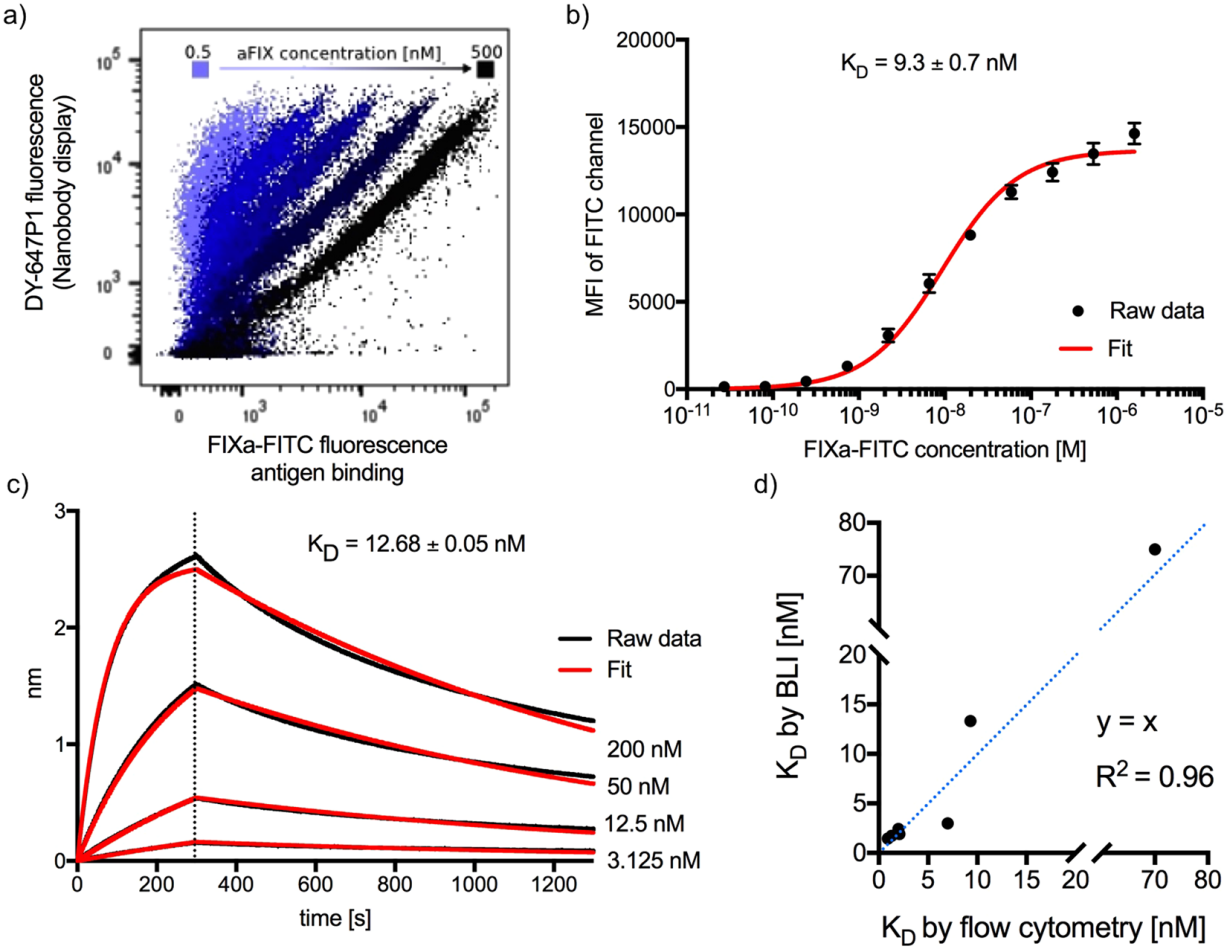
The binding affinities of Nanobodies that are displayed on yeast can conveniently be estimated by flow cytometry. (**a**) Yeast cells displaying one particular FIXa-specific Nanobody (MP1031_B7) were cultured overnight and orthogonally labelled with CoA-647, then divided in six aliquots and incubated separately with different concentrations of FIXa-FITC (0.5, 2, 8, 32, 128, 500 nM) and analysed in two dimensions by flow cytometry. (**b**) An apparent affinity of displayed Nanobody MP1031_B7 for the fluorescent antigen was determined by plotting the FITC mean fluorescence intensity (MFI) of Nanobody displaying cells versus the FIXa-FITC concentration. An apparent K_D_ (9.3 ± 0.7 nM) can conveniently be calculated by standard software like Prism 7 (GraphPad), using the simple one-site specific binding model. The data is shown as mean standard error of the mean (s.e.m.) from n = 3 independent experiments. (**c**) Association and dissociation isotherms of FIXa to Nanobody MP1031_B7. The Nb was immobilised on an Ni-NTA bio-sensor and the binding kinetics were monitored by bio‐layer interferometry (BLI) on OctetRED96 (ForteBio). The measured responses (red lines) were fitted to a monophasic 1:1 binding model (black lines). (**d**) Comparison of the apparent affinities of eight different Nanobodies as determined by flow cytometry (yeast displayed Nb-Aga2p-ACP fusion, y-axis) or BLI (subcloned and purified Nb, x-axis).

Affinity is a key property that affects the use of antibodies in many applications. Exploiting the flexibility of flow cytometry, we performed antigen dose response experiments on single yeast clones to determine an apparent affinity of the displayed Nanobody without the need for subcloning, expression and purification of the recombinant antibody^45^. As described above, cultures from individual yeast cells were stained orthogonally with Sfp and CoA-647 to monitor Nanobody display, then aliquoted and incubated with different concentrations with FIXa-FITC using a serial dilution in the pM - µM range and analysed by flow cytometry (Fig. 5a). For each antigen concentration, we next calculated the FITC MFI for ~10,000 cells displaying Nanobodies and plotted these values against the concentration of the fluorescent antigen to estimate an apparent affinity (Fig. 5b). For all displayed Nanobody-Aga2p-ACP fusions analysed by this approach, the calculated apparent affinities were in (low) nanomolar range (Supplementary Table 1).

To judge the reliability of this flow cytometric approach, we subcloned, expressed and purified eight of these FIXa-specific Nanobodies from the periplasm of *E*. *coli*^19^ and determined their binding kinetics by bio-layer interferometry (BLI, more details in Methods). Accordingly, the His6-tagged Nanobodies were immobilised on Ni-NTA biosensors and the binding characteristics to different FIXa concentrations were examined by analysing association and dissociation isotherms at different antigen concentrations on an Octet RED96 (Fig. 5c). Conveniently, the apparent affinities obtained by flow-cytometry correlate reasonably well with the affinities determined by BLI (Fig. 5d and Supplementary Table 1), justifying a quick prescreening of separate yeast clones by FACS at decreasing antigen concentrations as an effective approach for the identification of high affinity binders. More important, this validates screening protocols in which the antigen concentration is gradually decreased in subsequent rounds of selection for the selection of high affinity antibodies.

### Orthogonally labelled Nanobodies can be released from the yeast cell wall for biophysical and functional characterization

The functional or biophysical characterisation of antibodies often relies on the purification and the subsequent labelling of the antibody with a fluorescent dye or biotin, considerably complicating high throughput analysis in antibody discovery pipelines. Our display system overcomes this bottleneck. Indeed, the displayed fusion protein can orthogonally be coupled to a biotin moiety and functionally released from the yeast cell wall by reducing the disulphide bonds that link Aga2p to Aga1p with DTT. The biotinylated Nanbody-Aga2p-ACP fusion can be trapped with streptavidin for subsequent applications, such as BLI.

**Figure 6:**
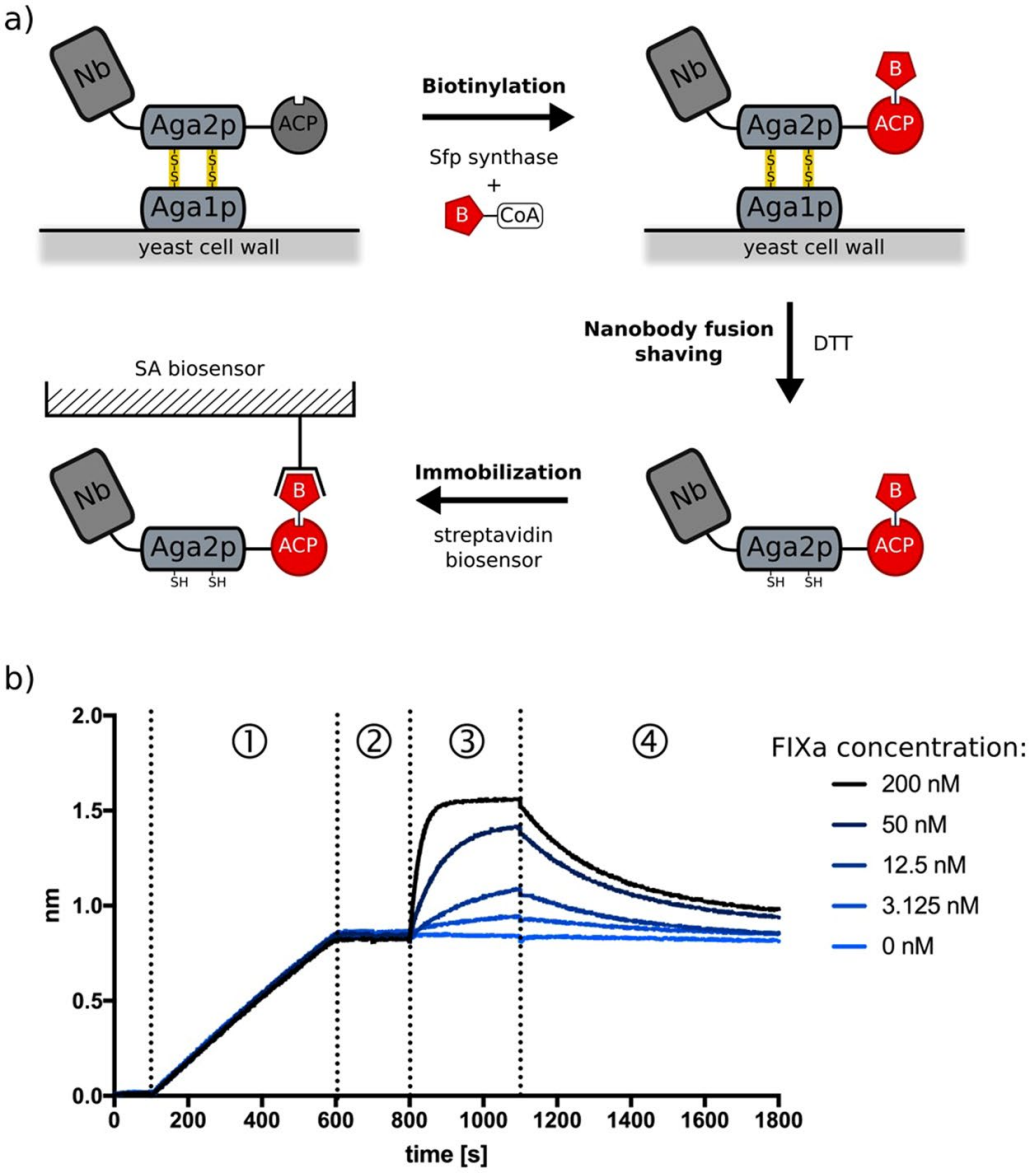
Orthogonally labelled Nanobodies can be functionally shaved from yeast cells and immobilised. (**a**) Nanobodies can be displayed on the yeast surface as Aga2p-ACP fusions and orthogonally biotinylated via ACP by adding a CoA-biotin derivative in the presence of Sfp synthase. Subsequent, this biotinylated Nanobody-Aga2p-ACP fusion protein can efficiently be shaved from the yeast surface by DTT and immobilised on streptavidin coated (SA) materials for other applications including BLI. (**b**) Binding isotherms (raw data) of the immobilisation of a biotinylated Nanobody-Aga2p-ACP fusion protein and the subsequent binding of its antigen to an SA-coated biosensor measured by BLI on OctetRED96. A 10 ml culture of yeast, displaying Nanobody MP1031_B7 as an Nb-Aga2p-ACP fusion was resuspended in with CoA-biotin and Sfp synthase and shaved by adding 2 mM DTT. A SA biosensor (ForteBio) was dipped into the supernatant (step 1) and equilibrated into buffer (step 2). Next, the biosensor was incubated with different FIXa concentrations to follow the association (step 3) and dissociation (step 4) of the antigen.

For example, we displayed FIXa specific Nanobody-Aga2p-ACP fusions on yeast. Next, we supplemented the cultures with Sfp synthase and CoA-biotin and washed the cells to remove the free CoA-biotin derivative. The biotinylated fusions (Nanobody-Aga2p-ACP-biotin) can then be shaved off from the yeast surface by adding 2 mM DTT and collected in the supernatant by centrifugation. In one application, streptavidin biosensors were applied to three such supernatants to capture the corresponding biotinylated Nanobodies and determine their on- and off-rates by BLI on Octet RED96 (Fig. 6). Supplementary Table 1 illustrates that the binding affinities of the unpurified Nanobody-Aga2p-ACP-biotin fusions that were shaved from the yeast cells are similar to those obtained from the corresponding His6-tagged Nanobodies that were subcloned and purified to homogeneity from the periplasm of *E*. *coli*. In addition to these straightforward binding measurements, other crucial antibody properties such as cross-reactivity to other proteins or homologs and possible inhibition of protein-protein interactions can be examined in a short time by using different BLI-based experimental setups. Long term storage of the active CoA-modified Nanobody-Aga2p-ACP fusion (Supplementary Fig. 2) is a remarkable improvement in the often multi-step strict antibody validation procedures.

### Selection of Nanobodies against the human α_2A_ adrenergic receptor

The discovery of antibodies that target integral membrane proteins remains a challenge. Accordingly, we also tested our Nanobody display/selection platform for the selection of Nanobodies directed against human α_2A_ adrenergic receptor (α_2A_AR)^46^, a G protein-coupled receptor. The recombinant receptor was purified from Sf9 insect cells and reconstituted in proteoliposomes in the presence of the covalent agonist DW38 to immunise a llama for generation of the yeast display library 192 (Lib192).

Three rounds of selection by yeast display and FACS were performed essentially as described above. Yeast cells containing Lib192 were grown, induced and stained orthogonally with CoA-647 using Sfp synthase to monitor the display density. Recombinant α_2A_AR was solubilised in DDM in the presence of antagonist RX821002 (Tocris) and labelled with the Alexa Fluor 488 (Alexa488) fluorophore by NHS chemistry. After labelling, the receptor was purified by gel filtration and the protein/fluorophore ratio was determined at 1.0 ± 0.1. In the subsequent rounds of selection, the concentration of fluorescent antigen was decreased from 500 nM (first round) to 100 nM (following rounds) to gradually enrich for yeast cells with a high display density and a high capacity to bind α_2A_AR-Alexa488 (Supplementary Fig. 5).

After three rounds of selection, the yeast cells that combined high fluorescence in the DY-647P1 and the Alexa488 channels were sorted, plated on selective medium and 96 individual clones were further characterised. Each clone was analysed by flow-cytometry for binding to 100 nM of the fluorescent receptor (α_2A_AR-Alexa488) and the sequence of the corresponding Nanobody was determined to identify ten unique Nanobody sequence families that bind α_2_AR. For one Nanobody from each sequence family, the apparent affinity for the receptor was determined by flow cytometry as described above, extending from 5 to 230 nM (Supplementary Table 1).

### Selection of Nanobodies against the human OX_2_ orexin receptor

The human OX_2_ orexin receptor was used as a next example in targeting GPCRs by using our new display/selection system. N-terminally FLAG-tagged and C-terminally His10-tagged OX_2_ orexin receptor (OX_2_R) was expressed in insect cells and purified to homogeneity in detergent MNG. Purified receptor bound to the OrexinB peptide was also reconstituted in proteoliposomes. A llama was immunised five times with detergent solubilised OX_2_ and boosted once with OX_2_R reconstituted in proteoliposomes for the generation of yeast display library 197.

Yeast cells displaying library 197 were grown, induced and stained orthogonally with CoA-647. OX_2_R was solubilised in MNG and labelled with a fluorescent anti-FLAG M1 monoclonal antibody with an Alexa488/mAb ratio of 2.7 ± 0.2 (M1 mAb-Alexa488). In three subsequent rounds of selection, the concentration of MNG-detergent solubilised OX_2_R, preincubated with a five-fold molar excess of M1 mAb-Alexa488, was decreased from 500 nM (first round) to 100 nM (following rounds). Control experiments in which the yeast cells were incubated with M1 mAb-Alexa488 alone were performed in parallel to monitor the undesired enrichment of Nanobodies that bind to the monoclonal antibody (Supplementary Fig. 5).

After three rounds of selection, 96 yeast colonies derived from gate Q2 (Supplementary Fig. 4) were analysed separately by flow-cytometry for binding to 100 nM OX_2_R in the presence of 300 nM of the fluorescent M1 mAb-Alexa488 (Supplementary Fig. 6). Nine Nanobodies from different sequence families were validated to bind specifically to OX_2_R but not to fluorescent mAb. The apparent affinity of these nine Nanobodies were measured by flow cytometry, ranging from 8 to 270 nM (Supplementary Table 1). Next, the OX_2_R-Nanobody complex formation was investigated by size-exclusion chromatography. Nanobodies validated by flow-cytometry were subcloned into WK6 cells, expressed and purified to homogeneity. For each Nanobody clone, the purified human OX2R was incubated with a three-molar excess of Nanobody and applied on a gel filtration column. Eluted fractions were analysed by SDS-PAGE confirming the OX2R-Nanobody complex formation for all nine selected clones (Supplementary Fig. 7).

## Conclusion

We developed an improved platform for the screening of affinity reagents by yeast surface display followed by flow cytometric analysis. By implementing orthogonal labelling of the surface exposed reagent, our system allows us to robustly monitor the display level on each individual yeast clone. Orthogonal labelling also enabled us to easily perform an initial biophysical or biochemical characterisation of the binders without the need to subclone, express or purify them from another host. We validated this system by selecting high affinity Nanobodies against one soluble and two membrane proteins.

### Ethics Statment

All animal vaccination experiments were executed in strict accordance with good animal practices, following the European Union animal welfare legislation and after approval of the local ethical committee (Committee for the Use of Laboratory Animals at the Vrije Universiteit Brussel). Every effort was made to minimize animal suffering.

## Methods

### Recombinant expression and purification of human coagulation factor FIX

FIXa was expressed and purified essentially as previously described^47^. Briefly, the recombinant protein comprises the EGF2 domain (as from Cys88) followed by the activation peptide and the catalytic domain, including the three point mutations (Y94F K98T and Y177T) that improve the biochemical properties of FIX^43^. This truncated FIX construct was cloned into a modified pET22b (+) vector (Novagen), lacking the pelB leader sequence and His-tag. Expression was carried out in BL21 (DE3) *E*. *coli* cells resulting in inclusion body formation. The protein was resolubilised in 8.5 M guanidine hydrochloride and refolded by rapid dilution, then dialysed and polished by ion-exchange on Q-sepharose. Concentrated FIX (2 mg/ml) was activated enzymatically with FXIa (Coachrom) used at a molar ratio of 200:1. After 1 to 2 days of incubation, full activation was confirmed by SDS-PAGE. Finally, activated FIX was covalently modified by adding a twenty-fold molar excess of the suicide inhibitor PPACK (Haematologic Technologies) or its fluorescent derivative FITC-PPACK (Haumatologic Technologies) for 4 h at RT and recovered on heparin sepharose. The FIXa-FITC labelling efficiency was estimated at 0.95 ± 0.06 by comparing the absorbance of the protein (280 nm) to the absorbance of the fluorophore (493 nm).

### Recombinant expression and purification of human α_2A_ adrenergic receptor

α_2A_AR was expressed in Sf9 insect cells and culture media was supplemented with 2 μM rauwolscine (Tocris) to stabilise the receptor during expression. Cells were infected at a density of 4 × 10^6^ cells per ml and expressed at 27 °C for 48 hours. After harvesting, cells were lysed in a buffer comprised of 10 mM Tris-HCl pH 7.5, 1 mM EDTA, 160 μg ml-1 benzamidine, 100 μg ml-1 leupeptin, 2 mg ml-1 iodoacetamide and 1 μM RX821002 (Tocris). The cell membranes were centrifuged at 10,000 *g* for 20 min at 4 °C. Receptor was extracted using a Dounce homogeniser with a buffer of 20 mM HEPES, pH 7.5, 0.5 M NaCl, 1% (w/v) n-dodecyl-b-D-maltopyranoside (DDM; Anatrace), 0.2% sodium cholate, 0.02% cholesteryl hemisuccinate (CHS), 160 μg ml-1 benzamidine, 100 μg ml-1 leupeptin, 2 mg ml-1 iodoacetamide and 1 μM RX821002, and stirred for 1 hour at 4 °C. After centrifugation at 10,000 *g* for 20 min at 4 °C, the supernatant was incubated with Ni-NTA agarose beads (GE Healthcare) with stirring for 2 hours at 4 °C. The receptor bound Ni-NTA agarose beads were transferred onto a gravity column, and the receptor was eluted in a buffer of 20 mM HEPES, pH 7.5, 0.5 M NaCl, 0.1% DDM, 0.02% sodium cholate, 0.002% CHS, 160 μg ml-1 benzamidine, 100 μg ml-1 leupeptin, 1 μM RX821002 and 250 mM imidazole. The eluted receptor was then loaded over anti-Flag M1 affinity resin and exchanged into a buffer of 20 mM HEPES, pH 7.5, 0.5 M NaCl, 0.1% MNG, 0.02% sodium cholate, 0.002% CHS, 10 μM RX821002. Following extensive washing, the receptor was eluted and loaded on a Superdex 200 size exclusion column (GE Healthcare) with a buffer containing 20 mM HEPES, pH 7.5, 0.1 M NaCl, 0.01% MNG, 0.002% sodium cholate, 0.0002% CHS, 10 μM RX821002.

For yeast display purposes, purified α_2A_AR was labelled with a fivefold molar excess of Alexa488-NHS ester (Thermo Fisher Scientific). After a 30 min incubation at room temperature and a 30 min incubation on ice, unreacted label was quenched with 50 mM Tris pH 8 and removed by an additional gel filtration step as described above. A protein/fluorophore ratio was determined at 1.0 ± 0.1 by comparing the absorbance of the protein (280 nm) to the absorbance of the fluorophore (495 nm).

### Recombinant expression and purification of human OX_2_ orexin receptor

A full-length DNA fragment of human OX_2_R was cloned into a modified pFastBac (Invitrogen) baculovirus expression vector with the HA signal sequence followed by a FLAG tag at the amino terminus. For purification, a deca-Histidine tag was added at the carboxyl terminus. The resulting construct was transfected into Sf9 cells to produce a recombinant baculovirus with the Bac-to-Bac system (Invitrogen). Sf9 cultures were infected with recombinant baculovirus at a cell density of 3 × 10^6^/ml. Infected cells were grown for 48 hours at 27 °C, and cells were harvested and stored at −80 °C for future use. Sf9 cells were lysed in a hypotonic buffer containing 10 mM Tris-HCl pH 7.4, 1 mM EDTA, 160 μg/ml benzamidine, 100 μg/ml leupeptin, 2 mg/ml iodoacetimide and ligand (1 μM Suvorexant, Selleckchem or 100 nM orexin peptide b, UT Southwestern Medical Center Protein Chemistry Technology Center). Lysed membranes were resuspended and homogenised by dounce in a buffer containing 50 mM Tris pH 7.4, 500 mM NaCl, 1% (w/v) n-dodecyl-β-D-maltopyranoside (DDM, Anatrace), 0.2% Na Cholate, 0.2% cholesteryl hemi-succinate (CHS), 10% glycerol, 2 mg/ml iodoacetamide and ligand (5 μM Suvorexant or 100 nM orexin peptide b). Solubilisation proceeded for 1 hour at 4 °C, followed by ultra-centrifugation for 30 min at 100,000 *g*. After centrifugation, the solubilised supernatant supplemented with 20 mM imidazole was incubated with Ni-NTA agarose beads (GE Healthcare) in batch-binding mode for 4 hours at 4 °C. After binding, beads were washed with 10 column volumes of Ni-NTA buffer: 50 mM Tris pH 7.4, 500 mM NaCl, 0.05% DDM, 0.01% Na Cholate, 0.01% CHS, 5% glycerol, 50 mM imidazole and ligand (5 μM Suvorexant or 100 nM orexin peptide b). Protein was eluted with 5 column volumes of Ni-NTA wash buffer with 200 mM imidazole. The eluate from Nickel-affinity chromatography was supplemented with 2 mM Calcium and loaded onto M1 anti-Flag affinity beads (Sigma). Detergent was exchanged on the M1 resin from DDM to 0.05% lauryl maltose neopentyl glycol (LMNG, Anatrace). Receptor was eluted from the M1 beads with buffer containing 200 μg/ml FLAG peptide plus 5 mM EDTA. Finally, protein was concentrated in a 100 kD-cutoff Vivaspin column (Sartorius) and run on a Superdex 200 size exclusion column (GE Healthcare).

### Yeast display vectors construction

pNACP (Fig. 2a,c) is a derivative of yeast surface display vector pCTCon2 (Addgene 41843). First, the HindIII restriction site within the Trp gene in pCTCon2 vector was removed using site-directed mutagenesis by Quick-Change (Stratagene, La Jolla, CA) using primers TU3 and TU4 (Supplementary Table 2). A new display cassette, encoding the appS4 leader sequence (LS)^40^, followed by a multi cloning site (MCS), followed by the Aga2p anchor protein and by the ACP and the Myc tag (Fig. 2) was synthesised (GeneArt Gene Synthesis, Thermo Fisher Scientific) and cloned as an EcoRI and BglII digested DNA fragment to replace the original cassette. pNS6 and pNSNAP were constructed similarly by introducing the corresponding synthetic display cassettes. pNACP, pNS6 and pNSNAP can be linearised efficiently by BamHI and HindIII to introduce single nanobody genes or to construct complete immune libraries by *in vivo* homologous recombination in yeast.

### Construction of Nanobody display libraries

To evoke immune response against three different antigens, we immunised one specific antigen per llama (three llamas in total). The antigen was injected six times in the presence of GERBU adjuvant LQ 3000 (GERBU Biotechnik) as described^19^. One llama was immunised with a total of 840 µg of PPACK-inhibited human FIXa, one llama with a total of 700 µg of purified human α_2A_AR and one llama with a total of 340 µg of purified human OX_2_ receptor. After the immunisation procedure blood samples were collected, peripheral blood lymphocytes (PBLs) were isolated and RT-PCR was performed as described^19^. RNA fragments encoding collections of Nanobodies derived from the *in vivo* matured heavy chain–only antibody repertoire were amplified as follows. Total RNA was isolated from the PBLs to prepare cDNA and the open reading frames encoding all immunoglobulin heavy-chains were amplified by RT-PCR with primers call001 and call002^19^. Nanobody open reading frames were amplified thereafter through a nested PCR using primers TU1 and TU58 (Supplementary Table 2) to generate flanking sequences amenable to homologous recombination into pNACP. Accordingly, PCR products (10 µg) were mixed with BamHI/HindIII linearised pNACP vector (10 µg) and introduced into electrocompetent EBY100 cells as described^48^.

### Coenzyme A derivatives

CoA-biotin and fluorescent CoA-547, CoA-647 derivatives were purchased at New England BioLabs. CoA-Alexa488 was synthesised essentially as described before^35^, with Alexa Fluor^TM^ 488-C_5_ Maleimide (Thermo Fisher Scientific) and Coenzyme A sodium salt hydrate (Sigma).

### Orthogonal staining of Nanobody immune libraries displayed on yeast

Yeast cells displaying a Nanobody by use of our optimised system (Fig. 2) were inoculated in SDCAA medium (20 g glucose, 6.7 g yeast nitrogen base, 5 g bacto casamino acids, 5.4 g Na_2_HPO_4_ and 8.56 g NaH_2_PO_4•_H_2_O _dissolved_ in 1 litre of H_2_O, supplemented with 10 ml of Gibco^TM^ Penicinillin-Stereptomycin, 10,000 U/ml Thermo Fisher Scientific) and grown to an OD_600_ of 1. When displaying libraries, the inoculum contained at least ten times more cells than the library size. For induction, cells were harvested at 4,000 *g* and resuspended in SGCAA medium (20 g galactose, 6.7 g yeast nitrogen base, 5 g bacto casamino acids, 5.4 g Na_2_HPO_4_ and 8.56 g NaH_2_PO_4•_H_2_O in 1 litre of H_2_O, supplemented with 10 ml of Gibco^TM^ Penicinillin-Stereptomycin, 10,000 U/ml Thermo Fisher Scientific) and grown at 30 °C overnight at 120 rpm. For orthogonal staining, a total of 4 × 10^7^ induced yeast cells were washed twice in cold PBS supplemented with 0.2% (w/v) BSA at pH 7.4 (PBS–BSA), harvested at 4,000 *g* and stored on ice. For the covalent labelling of ACP, cells were routinely resuspended in 100 µl of reaction buffer (50 mM HEPES, pH 7.4, 10 mM MgCl_2_) supplemented with 1 µM of the Sfp synthase and 1 µM of the CoA derivative of choice: CoA-547 fluorophore, CoA-647 fluorophore, CoA-biotin (New England BioLabs) or synthesised CoA-Alexa488. After 60 min of staining in a rotating 50 rpm at room temperature, the labelled cells are washed three times with 500 µl of ice-cold PBS–BSA and can be stored for days on ice.

### Confocal microscopy

Yeast cells, displaying Nb35 were induced, next orthogonally stained with CoA-547 as described above and prepared for confocal microscopy imaging as described before^49^. Cells were observed under a laser scanning confocal microscope (Nikon Eclipse TE2000-U) with excitation wavelength of 543 nm and 100-fold magnifying objective.

### Nanobody selections by yeast display and FACS

Yeast display libraries Lib191 (FIXa), Lib192 (α_2A_AR) and Lib197 (OX_2_R) were inoculated, induced and orthogonally stained with CoA-647 as described above. In each round of selection, 4 × 10^7^ CoA-647 stained yeast cells were incubated for 60 min at 4 °C (rotating at 50 rpm) with the corresponding fluorescent antigen in 500 µl of an ice-cold antigen-specific buffer: 10 mM HEPES pH 8.0, 300 mM NaCl, 2.5 mM CaCl_2_, 0.2% BSA for FIXa (FIXa FACS buffer); 20 mM HEPES pH 7.5, 100 mM NaCl, 0.01% MNG, 0.002% CHS + 1 μM RX82 for α_2A_AR (α_2A_AR FACS buffer); 20 mM Tris pH 7.4, 150 mM NaCl, 0,025% LMNG, 0.001% CHS, 1 μM OrexinB peptide for OX_2_R (OX_2_R FACS buffer). After incubation with the fluorescent antigens, yeast cells were washed three times with the specified FACS buffer and resuspended in 2 ml final volume for sorting on a FACS Aria (BD Biosciences). Selected yeast cells were sorted into SDCAA medium, grown and induced, then stained again for consecutive rounds of selection. Aliquots of the sorted yeast cells were routinely plated in parallel as single colonies on SDCAA agar plates, picked and grown into 500 μl SDCAA medium in 96-well plates for further characterisation. For sequencing, 5 × 10^5^ yeast cells were incubated for 60 min at 37 °C in 10 μl of lysis buffer (10 mM Tris pH 7.4, 50 mM KCl, 1.5 mM MgCl_2_,) supplemented with Lyticase (500 U/ml, Sigma) followed by one freeze-thaw cycle to use 2.5 µl as the template to amplify the Nanobody-encoding DNA fragments by PCR with primers TU19 and TU20 (Supplementary Table 2).

### Nanobody screening and affinity determination by flow cytometry

Sorted yeast clones were grown separately in 96-well plates and induced in 500 µl SGCAA. Cells displaying a well-characterized Nanobody that binds an irrelevant antigen were included systematically as a negative control. For flow cytometry analysis of individual clones, 10^6^ cells were routinely transferred to a fresh 96-well plate, washed and stained orthogonally with CoA-647 in a final volume of 15 µl as described above. 10^5^ of these stained cells were mixed with 100 nM of the fluorescent antigen in 50 µl of FACS buffer (see above) and incubated for 60 min at 4 °C with shaking 50 rpm. To remove unbound fluorescent antigen, cells were washed two times with FACS buffer and applied on a FACS Fortessa (BD Biosciences). Routinely, 10,000 yeast cells of each well were analysed using FlowJo software (FlowJo, LLC) and compared to the negative control.

To determine the apparent affinity of individual Nanobodies displayed on yeast by flow cytometry we followed a similar procedure except that aliquots of the same clone distributed into 96-well plate and incubated for 60 min with serial dilutions (µM - pM range) of the fluorescent antigen. For each antigen concentration, the MFI of 10,000 yeast cells was calculated and plotted against the concentration of the fluorescent antigen. An apparent K_D_ was calculated by Prism 7 (GraphPad) using the one-site specific binding model.

### Nanobody subcloning and expression in *E*. *coli*

The open reading frames of Nanobodies selected by yeast display can conveniently be amplified by PCR from Lyticase treated yeast cells using primers EP229 and EP230 (Supplementary Table 2) and cloned as a SapI digested fragment in a Golden Gate variant of pMESy4 (GenBank KF415192). Nanobodies containing a C-terminal His6-tag followed by the EPEA-tag^7^ were routinely expressed in and purified from the periplasm of *E*. *coli* strain WK6^19^. Cells were grown in Terrific Broth medium supplemented with Ampicillin (100 mg/ml) and glucose (0.1% w/v) at 120 rpm and 37 °C to OD_600_ = 0.8 and induced overnight with 1 mM IPTG at 28 °C. The next day, cells were harvested by centrifugation (5,000 *g*, 15 min) and the periplasmic fraction was extracted using an osmotic shock (Pardon 2014). After separation from the protoplasts by centrifugation (5,000 *g*, 30 min) the clarified supernatant was loaded on a HisTrap FF 5 mL prepacked column. The Nanobody was eluted from the NiNTA resin by applying 500 mM imidazole and concentrated by centrifugation using Nominal Molecular Weight Limit (NMWL) filters (Sigma) with a cut-off of 3 kDa.

### Antigen binding kinetics of purified Nanobodies

For eight purified Nanobodies, we determined the binding kinetic parameters for FIXa by OctetRED96 (ForteBio). Purified His6- and EPEA-tagged Nanobodies were diluted to 2 µg/ml in 10 mM HEPES pH 8.0, 300 mM NaCl, 2.5 mM CaCl_2_, 0.5% BSA, 0.04% Tween 20 and directly immobilised on NiNTA biosensors at about 1 nm response. After an equilibration step of 100 s, the binding isotherms were monitored by exposing separate sensors simultaneously to different concentrations of FIXa. The association of the antigen was measured for 300 s, tailed by a dissociation experiment for 900 s.

All kinetic experiments were performed at 30 °C under constant stirring at 1000 rpm. A biosensor that was exposed buffer alone was used to monitor the background of the sensograms. Association and dissociation rates were calculated by fitting to sensograms using the 1:1 binding model included in the Octet Data Analysis software 9.1 (ForteBio).

### Antigen binding kinetics of biotinylated and shaved Nanobodies

Three biotinylated Nanobodies were released from the yeast cell wall with DTT (shaved) and their binding kinetics measured by OctetRED96 (ForteBio) as follows. Yeast cells displaying a Nanobody-Aga2p-ACP fusion were grown and induced in SGCAA. 4 × 10^8^ induced cells were washed two times with PBS-BSA and stained orthogonally with CoA-biotin in 1 ml final volume, then washed three times and shaved by adding PBS containing 2 mM DTT for 30 min at room temperature to reduce the disulphide bonds between Aga1p and Aga2p. After centrifugation (4,000 *g*, 3 min), the supernatant was recovered and used to load the biotinylated Nanobody-Aga2p-ACP fusions on streptavidin biosensors at about 1 nm response. After an equilibration step of 100 s, the binding isotherms were monitored by exposing separate sensors simultaneously to different concentrations of FIXa. The association of the antigen was measured for 300 s, tailed by a dissociation experiment for 900 s.

### OX_2_R -Nanobody complex formation analysis by size exclusion chromatography

Purified human OX_2_R was mixed with a three-molar excess of the purified Nanobody and incubated for 60 min at 4 °C. Protein mixtures were loaded on a Superdex 200 10/300GL size-exclusion column (GE Helthcare), equilibrated with 20 mM Tris pH 7.4, 150 mM NaCl, 0.05% LMNG, 0.01% CHS, 100 nM orexin peptide b buffer. Eluted fractions were loaded on a 12.5% gradient SDS-PAGE gel.

## Supplementary information

Supplementary Information
