# Supplementary material for: An improved yeast surface display platform for the screening of nanobody immune libraries
Source: Sci Rep. 2019 Jan 23;9:382. doi: 10.1038/s41598-018-37212-3 (PMC6344588; doi:10.1038/s41598-018-37212-3)
Supplement: Supplementary file 1 — Supplementary Information [file 41598_2018_37212_MOESM1_ESM.pdf]

SUPPLEMENTARY INFORMATION

An improved yeast surface display platform for the screening of nanobody immune libraries.

Tomasz Uchański[1,2], Thomas Zögg[1,2], Jie Yin[3], Daopeng Yuan[4], Alexandre Wohlkönig[1,2], Baptiste Fischer [1,2], Daniel M. Rosenbaum[3], Brian K. Kobilka[4,5], Els Pardon[1,2], Jan Steyaert[1,2*].

AFFILIATIONS

[1] Structural Biology Brussels, Vrije Universiteit Brussel (VUB), Brussels, Belgium.

[2] VIB-VUB Center for Structural Biology, VIB, Brussels, Belgium.

[3] Department of Biophysics, The University of Texas Southwestern Medical Center, Dallas, Texas 75390, USA.

[4] Beijing Advanced Innovation Center for Structural Biology, Tsinghua-Peking Joint Center for Life Sciences, School of Medicine, Tsinghua University, Beijing 100084, China.

[5] Department of Molecular and Cellular Physiology, Stanford University School of Medicine, Stanford, California 94305, USA

* Corresponding author

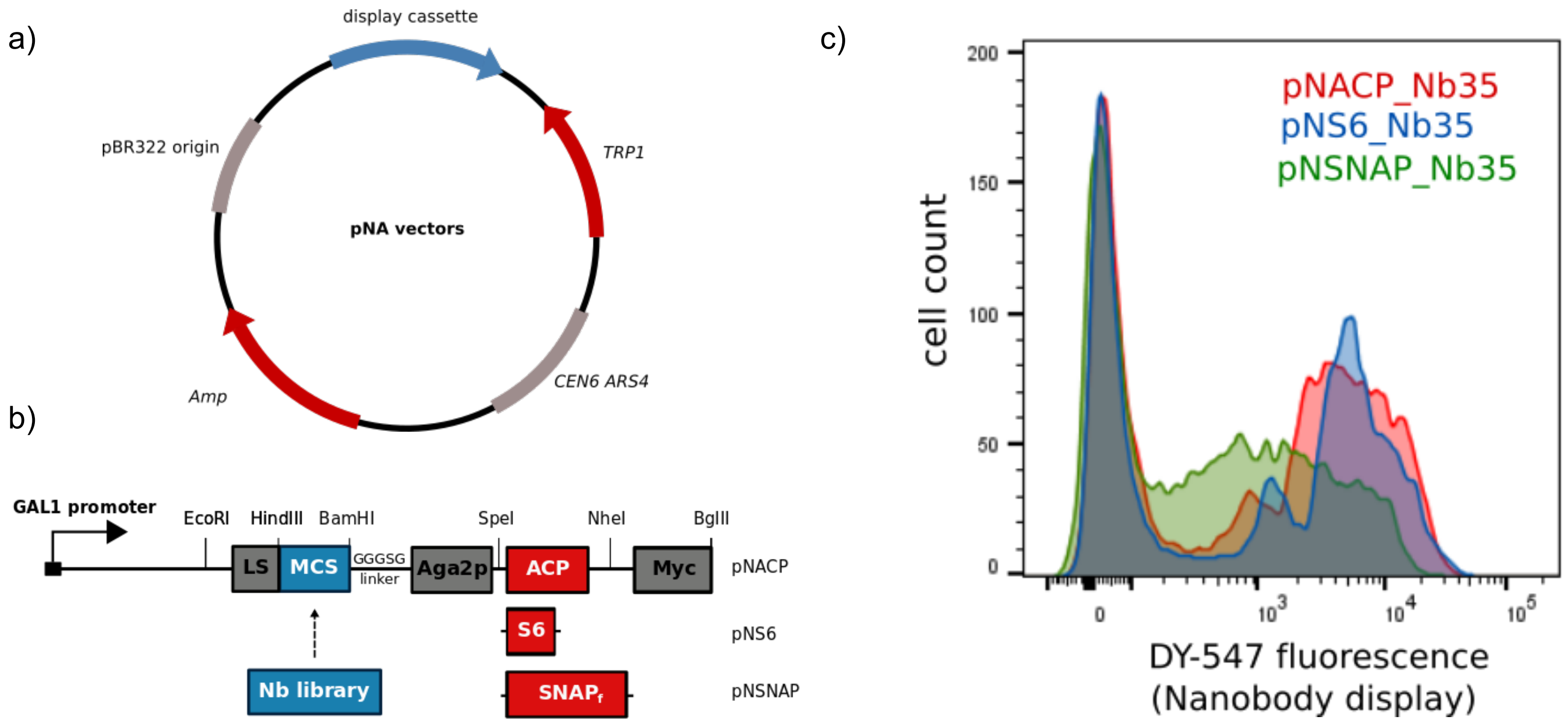

Supplementary Figure 1. Expression vectors for the display and orthogonal labeling of Nanobodies on the surface of yeast. (a) General overview of the versatile shuttle vectors that drive the inducible display and orthogonal staining of Nanobodies on the surface of yeast: *TRP1,* a phosphoribosylanthranilate isomerase gene that allows the selection of transformed EBY100 yeast cells; pBR322 origin, the *E. coli* origin of replication from pBR322; *Amp*, a bacterial ampicillin resistance gene; *CEN6 ARS4*, a yeast origin of replication. (b) Display cassettes encoding the ACP, S6 and SNAP fusion proteins respectively. The GAL1 promotor regulates an expression and the appS4 leader sequence drives the secretion of these fusion proteins in yeast. (c) EBY100 yeast cells containing pNACP-Nb35, pNS6_Nb35 or pNSNAP_Nb35 were grown and induced overnight in galactose-rich medium. The Nb35-Aga2p-ACP and the Nb35-Aga2p-S6 fusions were orthogonally labeled by incubating these cells with CoA-547 in the presence of Sfp synthase. The Nb35-Aga2p-SNAP fusion was orthogonally labeled in self-labelling reaction with SNAP-Surface 549 (New England BioLabs), fluorescence benzyl-guanine derivative. Yeast display level and labelling efficiency of CoA-547 labelled pNACP_Nb35 (red), pNS6_Nb35 (blue) and SNAP-Surface 549 labeled pNSNAP_Nb35 (green) displayed fusion proteins were compared by flow cytometry. The fluorescence intensity of each cell was monitored at 582 nm upon excitation with a 561 nm yellow-green laser.

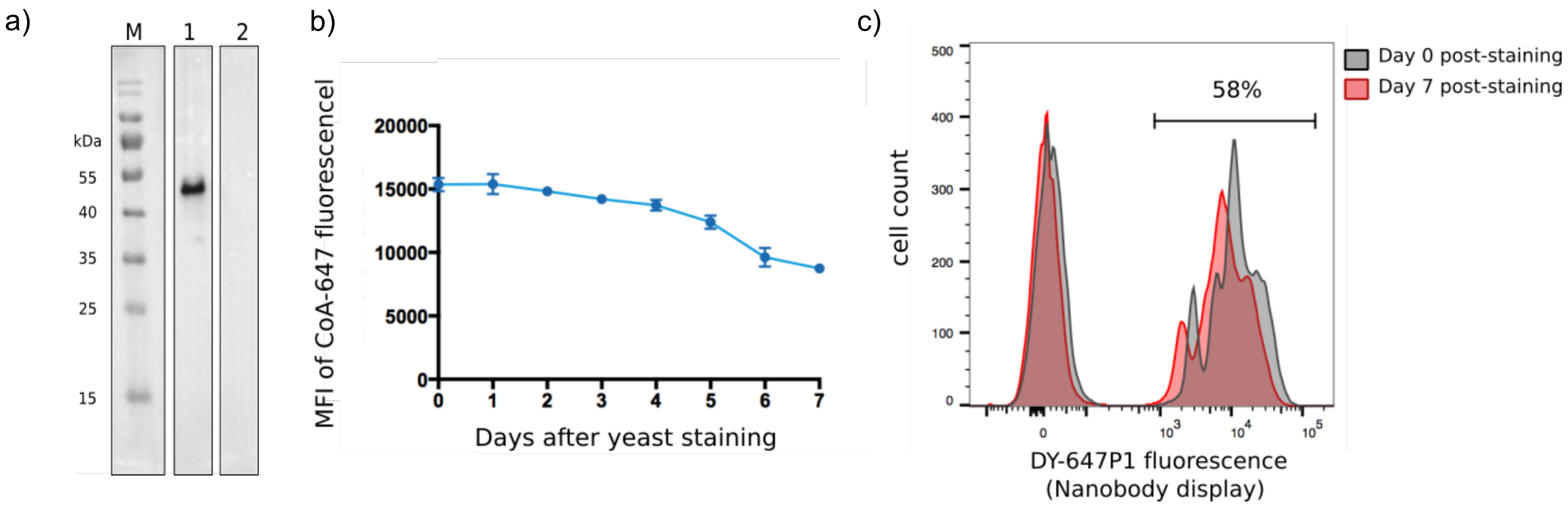

Supplementary Figure 2. Stability of the Nanobodies displayed on yeast surface. EBY100 yeast cells, bearing the pNACP_Nb35 and control cells containing the empty cloning vector pNACP, were grown and induced overnight in a galactose-rich medium. (**a**) To monitor the resistance of the displayed fusion protein to proteolytic degradation, 10^7 cells of each culture were harvested and incubated in PBS containing 20 nM of DTT for 30 min to release the Nb-Aga2p-ACP fusion protein from the yeast cell wall. The supernatants were subjected to western blot analysis using a mouse anti-c-myc tag mAb Y69 (Roche) followed by goat anti mouse-AP mAb (BioRad): line M, protein MW marker; line 1, yeast cells displaying Nb35-Aga2p-ACP-cmyc; line 2: control cells. (**b**) Induced cells were orthogonally labeled with CoA-647 and Sfp synthase and stored for one week in the dark at 4 °C. Every day, an aliquot was subjected to flow cytometry to monitor the stability of the labelled fusion proteins on a FACS Fortessa by measuring the CoA-647 fluorescence. **c**) Histograms of the CoA-647 fluorescence of labelled yeast cells at time 0 and 7 days post-staining.

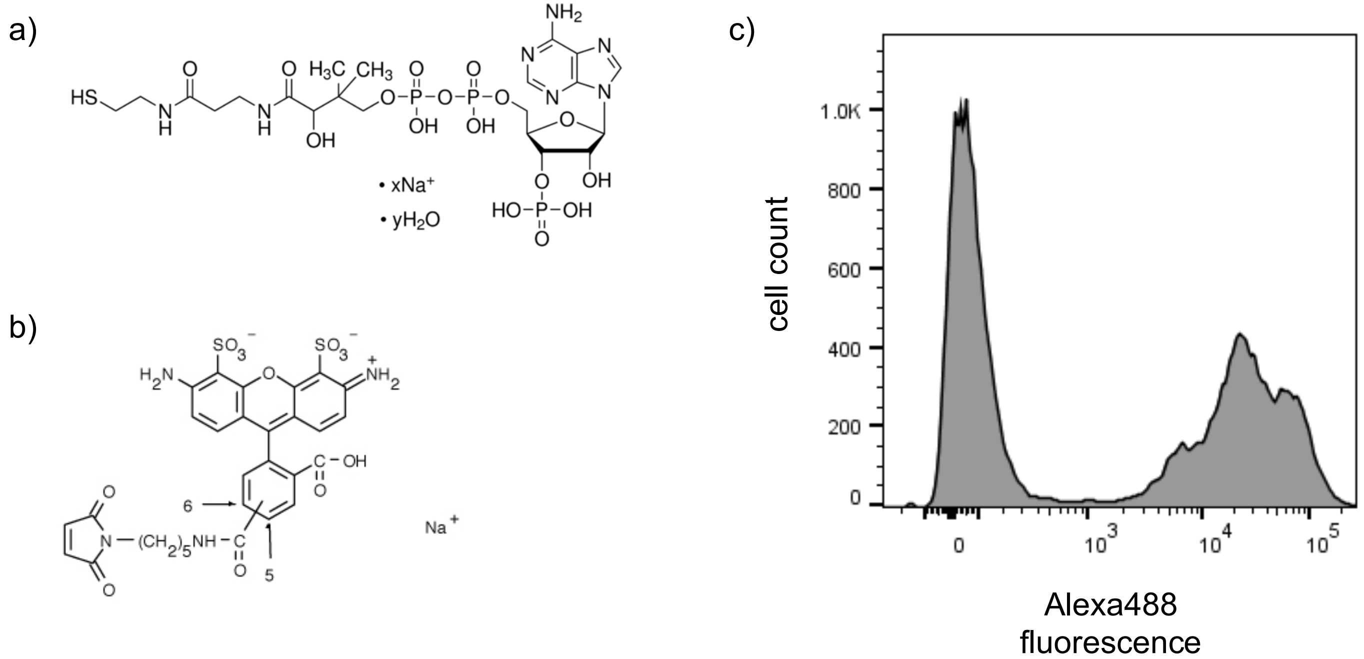

Supplementary Figure 3. Generation and validation of CoA-Alexa488. Commercially available Alexa Fluor^{TM} 488-C_5 Maleimide (a) from Thermo Fisher Scientific and Coenzyme A sodium salt hydrate (b) from Sigma were used for synthesis of the CoA-Alexa488 following synthesis procedure[35]. EBY100 yeast cells, bearing the pNACP_Nb35 and control cells containing the empty cloning vector pNACP, were grown and induced overnight in a galactose-rich medium. To validate the utilisation of the synthesised CoA-Alexa488 dye, yeast cells were stained as described above and analysed by flow cytometry (FACS Fortessa). **c**) Histograms of the Alexa488 fluorescence of labelled yeast cells.

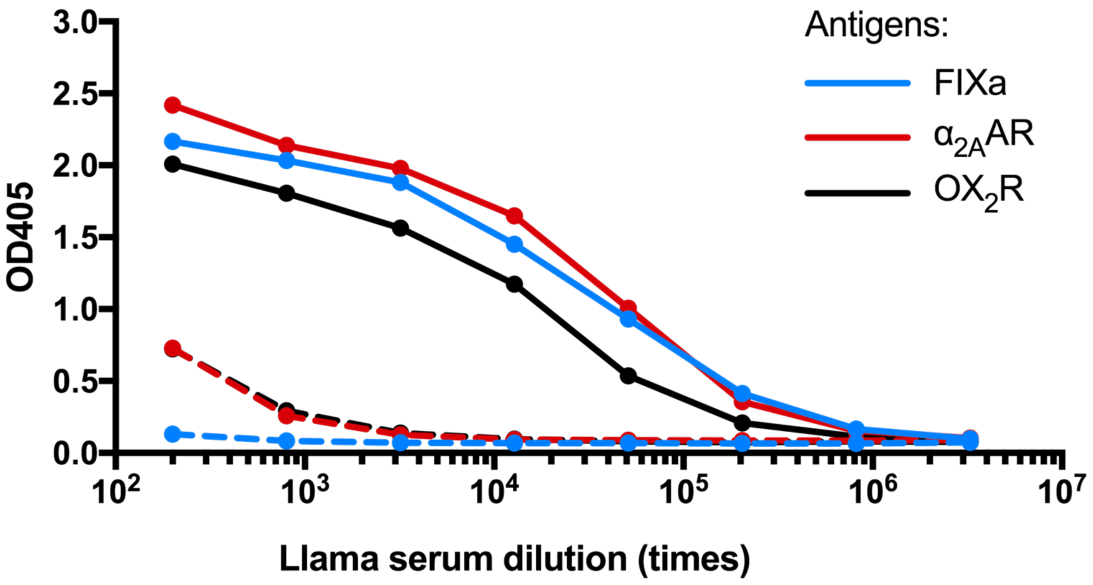

Supplementary Figure 4. Serum ELISA to monitor the Llama's humoral response after immunisation with FIXa, α_{2A}AR or OX_2R. Three different llamas were immunised separately with human α_{2A} adrenergic receptor (α_{2A}AR), human OX_2 orexin receptor (OX_2R) and coagulation Factor IX (FIXa). Blood sera were collected from each animal before (dashed lines) and after immunisation (solid lines) to confirm the induction of an antigen-specific immune response by serum-ELISA.

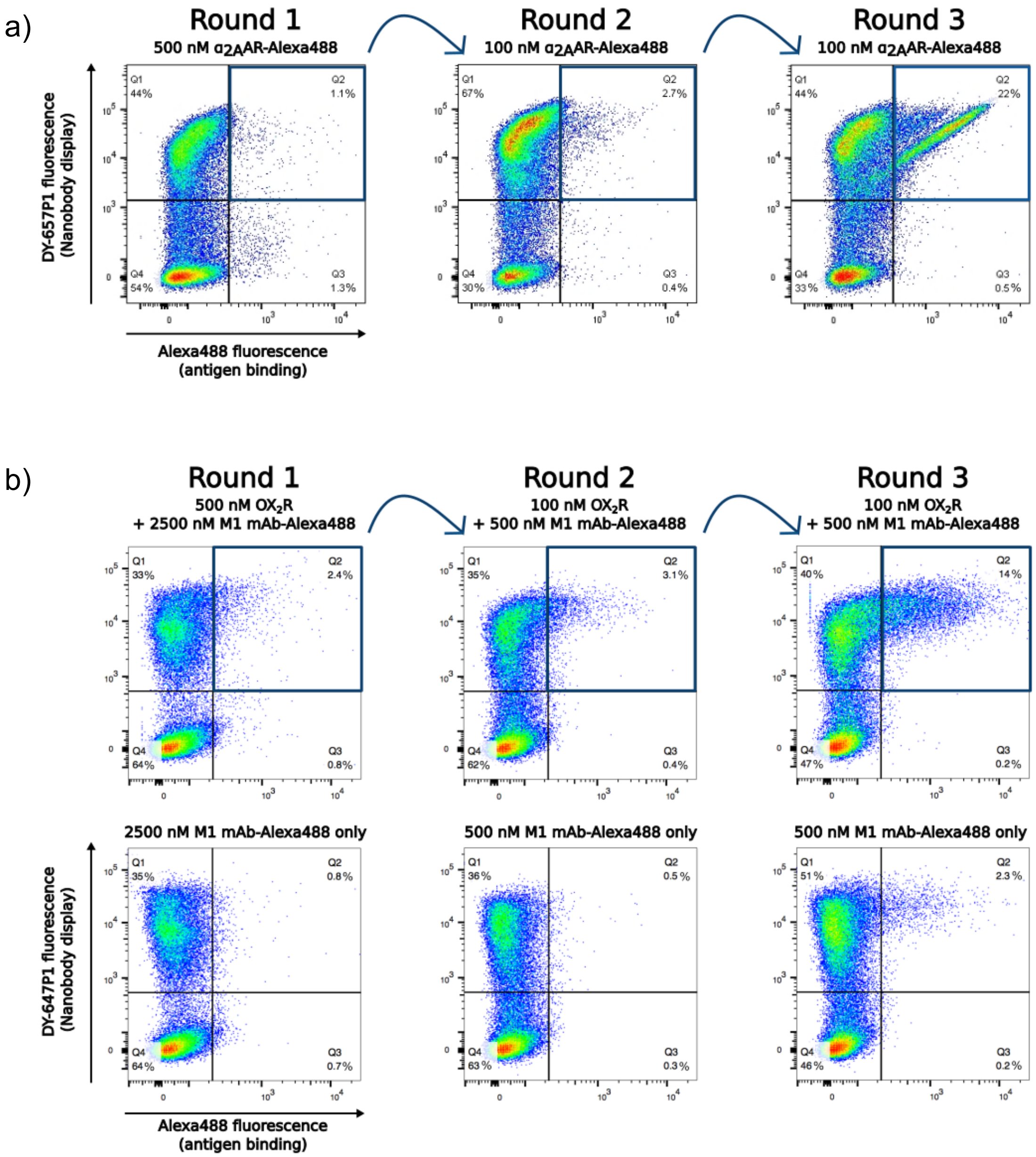

Supplementary Figure 5. Discovery of α_{2A}AR and OX_2R specific Nanobodies by 3 rounds of yeast display followed by FACS. Each dot represents two fluorescent signals of a separate yeast cell of the (sub)library. The Alexa488 fluorescence (x-axis) is a measure of the amount of the fluorescent antigen that is captured on the surface of the yeast cell. The CoA-647 fluorescence (y-axis) gives an indication of the Nanobody display level on each cell. (**a**) Dot plots of three consecutive rounds of selection by yeast display of library 192 using decreasing amounts of fluorescent α_{2A}AR-Alexa488, leading to a stepwise enrichment of antigen binding yeast cells (cells counted in gate Q2 represented as a blue square) from 1.1% in the first round of selection to 22% in the third round. (**b**) Dot plots of three rounds of selection by yeast display of library 193 using decreasing amounts of OX_2R stained with M1 mAb-Alexa488, leading to a stepwise enrichment of antigen binding yeast cells (cells counted in gate Q2 represented as a blue square) from 2.14% in the first round of selection to 14% in the third round. In parallel, each (sub)library was incubated with M1 mAb-Alexa488 alone as a control sample.

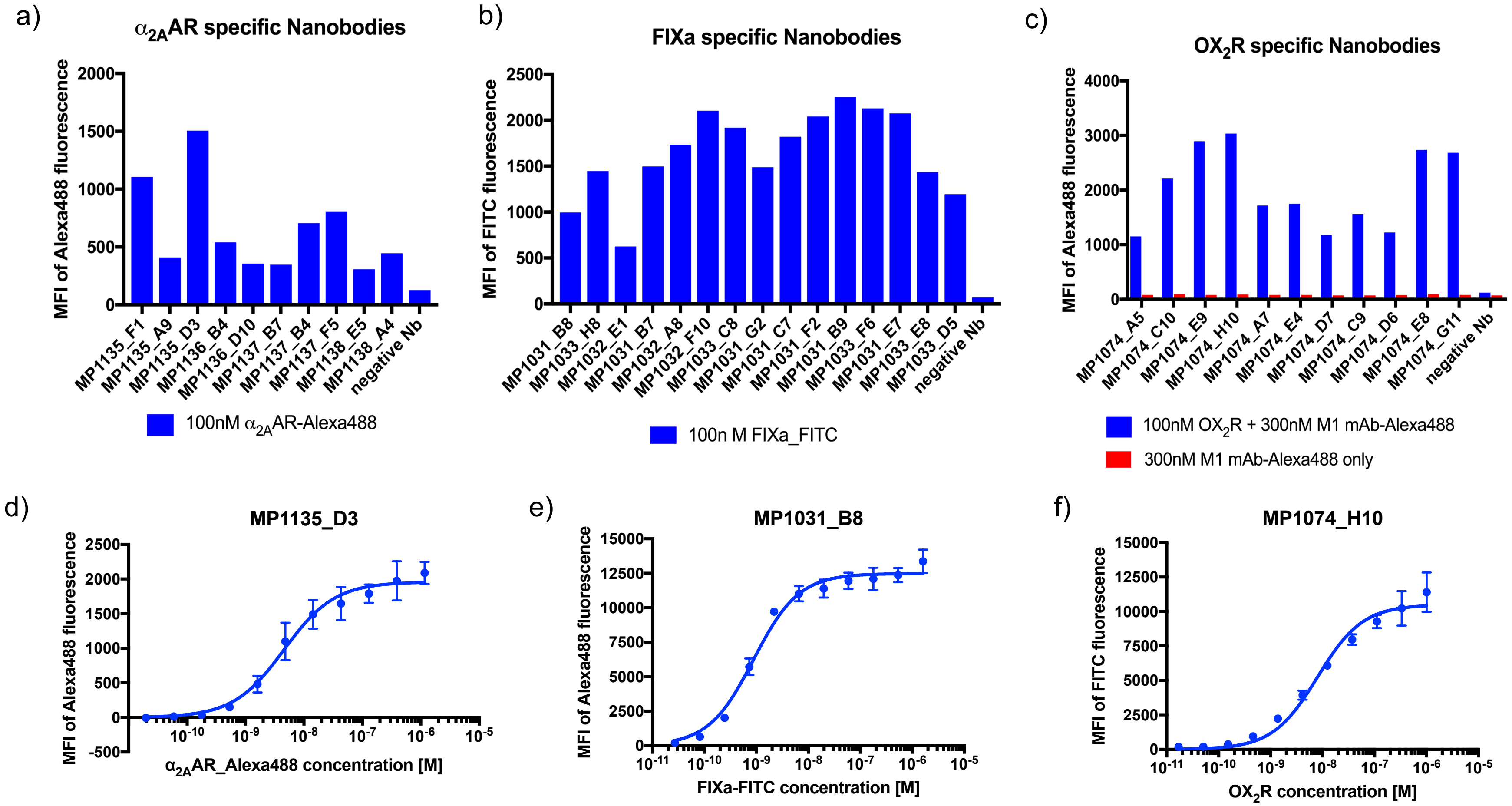

Supplementary Figure 6. Flow cytometric screen and apparent affinity determination of individual yeast cells displaying an antigen-specific Nanobody by FACS Fortessa. After selection, individual yeast cells were grown, induced overnight in 96-well format and orthogonally stained with CoA-647 to discriminate Nanobody-displaying cells from cells with a low Nanobody expression level. These cells were next incubated with the corresponding antigens and analysed by FACS Fortessa: a) 100nM of α_2AR-Alexa488, b) 100nM FIX-FITC and c) 100nM OX_2R labelled with 300nM M1 mAb-Alexa488. Using FlowJo software, the mean fluorescent intensities (MFI) of the antigen fluorescence was calculated for those yeast cells that display Nanobodies (coA-647 fluorescence above a preset threshold). In order to determine an apparent affinity each individual clone, stained yeast cells were incubated with different concentration of the cognate antigens and analysed as above. Titration curves of clones MP11355_D3 (d), MP1031_B8 (e) and MP1074_H10 (f) are the representative samples for α_2AR-Alexa488, FIXa-FITC and OX_2R respectively. The data is shown as mean standard error of the mean (s.e.m.) from n = 3 independent experiments.

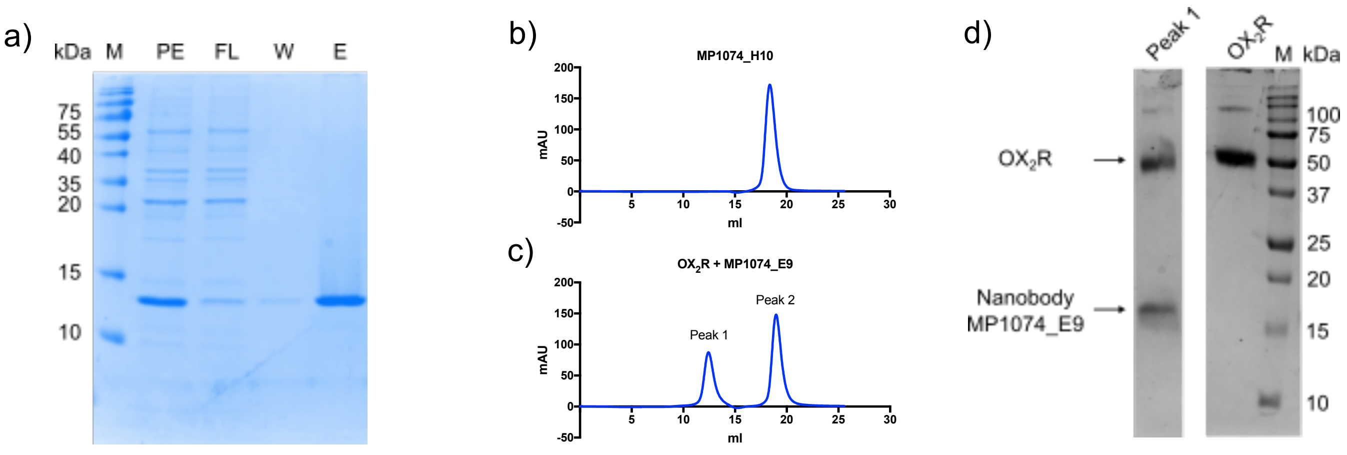

Supplementary Figure 7. Nanobody purification and complex formation with human OX_2 orexin receptor. a) SDS-PAGE analysis of the purification of the Nanobody MP1031_F2 by Ni-NTA affinity chromatography. Line M, protein MW marker; line PE, periplasmic fraction of WK6 cells expressing Nanobody; line FL: flow-through of the Ni-NTA resin; W, wash with 10 mM imidazole; E, elution with 500 mM imidazole. 10 µl of each fraction was loaded on a 15 % SDS-PAGE gel. b) Gel filtration profile (Superdex 200 PG 10/300GL) of the Ni-NTA purified Nanobody MP1074_H10. c) Gel filtration profile (Superdex 200 PG 16/90) of the purified OX_2R preincubated with a three-molar excess of Nanobody MP1074_E9. Peak 1 was further analysed on a 12.5 % SDS-PAGE gel (d). Comparison of the high molecular weight fraction (Peak 1) to sample of the purified OX_2R only (line OX_2R) confirms the tight OX_2R-Nanobody complex formation.

**Supplementary Table 1. Apparent K_D values of selected antigen-specific Nanobodies**			
**Nanobody**	**K_D by flow cytometry [nM][a]**	**K_D by BLI [nM][b]**	
	**Nanobody-Aga2p-ACP**	**Nanobody-His6**	**Nanobody-Aga2p-ACP-biotin**
**human coagulation factor IX**			
MP1031_F2	0.9 ± 0.1	1.46 ± 0.02	1.26 ± 0.02
MP1031_B7	9.3 ± 0.7	12.68 ± 0.05	12.5 ± 0.1
MP1033_D5	0.9 ± 0.1	0.83 ± 0.01	1.96 ± 0.03
MP1032_H10	7 ± 2	2.98 ± 0.02	
MP1033_H8	2.0 ± 0.3	2.42 ± 0.02	
MP1032_A8	71 ± 6	75.3 ± 0.2	
MP1032_F10	2.1 ± 0.3	1.92 ± 0.01	
MP1031_G2	1.3 ± 0.2	1.74 ± 0.02	
MP1031_C7	97 ± 11		
MP1032_E1	75 ± 13		
MP1031_B9	23 ± 4		
MP1033_E8	15 ± 1		
MP1033_C8	0.8 ± 0.2		
**human α_{2A} adrenergic receptor**			
MP1135_F1	89 ± 4		
MP1135_A9	104 ± 22		
MP1135_D3	4.5 ± 0.6		
MP1136_B4	63 ± 8		
MP1136_D10	124 ± 13		
MP1137_B7	5.1 ± 0.4		
MP1137_B4	57 ± 12		
MP1137_F5	223 ± 55		
MP1138_E5	165 ± 22		
MP1138_A4	172 ± 20		
**human OX_2 orexin receptor**			
MP1074_A5	25 ± 4		
MP1074_C10	186 ± 36		
MP1074_E9	268 ± 52		
MP1074_H10	8 ± 1		
MP1074_A7	85 ± 10		
MP1074_E4	64 ± 14		
MP1074_D7			
MP1074_C9	139 ± 25		
MP1074_D6	47 ± 8		
MP1074_E8	269 ± 30		
MP1074_G11			

[a] Values shown as mean ± standard error of the mean (s.e.m.) from *n* = 3 independent experiments. [b] Values shown as mean ± standard error of the mean (s.e.m.) from *n* = 1 experiment.

Supplementary Table 2. Primer sequences	
Primer #	sequence 5' - 3'
TU1	CAATTAGATAAAAGAGAGGCCGAAGCTCAGGTGCAGCTGGTGGAGTCTGGGG
TU3	CCAGCTAACATAAAATGTAAACTTTCGGGGCTCTCTTGCC
TU4	GGCAAGAGAGCCCCGAAAGTTTACATTTTATGTTAGCTGG
TU19	AACGTCAAGGAGAAAAAACC
TU20	AAGTACAGTGGGAACAAAG
TU58	CCCCTCCACCAGAGCCACCTCCGGATCCGCTGGAGACGGTGACCTGGGTCCC
TU62	TGCCTTGCCATTATCCGAT
EP229	CCTTGAGCTCTTCGGCCCAGGTGCAGCTGGTGGAGTCTGG
EP230	AGGACTGCTCTTCCACTGGAGACGGTGACCTGGGT

Supplementary Table 3. Amino acid sequences of Nanobodies, according to the IMGT® numbering.

Nanobody	CDR1	CDR2	CDR3
**human coagulation factor IX**			
MP1031_F2	GFTFDDYV	ISHIDGSI	AAEVDIAYSCLSADDYEI
MP1031_B7	GRTFSSYI	ISLSGGST	AASLKYGSTWYTLESYDY
MP1033_D5	GFTFDDYA	ISIGDGST	AAEFYVDCVLVGTNGGSADFES
MP1032_H10	GRSVSDYD	ISPNGGIT	AAGKGFTLRALAERRYDY
MP1033_H8	GRTMSAFR	MFSSSTGA	AARTLATNYSNTQPGGYDY
MP1032_A8	GIVFDDFA	ISSRDGFT	AADFGISCSEYDLGNPLTTEDYEA
MP1032_F10	ERTFKSYA	VRTDGST	APKVYSGGFFDIGAYDR
MP1031_G2	GRTVSMDN	ISAPRGFKT	AKKRGNWGIGTSAEYEYED
MP1031_C7	GRTFDNYA	IDWRGSST	ATGDLAMSVMKNARY
MP1032_E1	GRTFNNYA	INWNGGRT	AADRQSSAYVRSDNAY
MP1031_B9	GITFDAYD	IGENDGSI	ATDPDYYCSEYGPGGISLYHNELEY
MP1033_E8	ESAYV	ITRAGTE	YAGSIYNRHAY
MP1033_C8	GFTFHEHV	ISHIDGSI	AAELDIEGSCLKADEYEI

Appendix. Full sequence of the pNACP vector:
AmpR: 715-1575
GAL1 promoter: 2940-3606
appS4 Leader Sequence: 3644-3910
Multi Cloning Site: 3906-3943
Flexible linker: 3944-4045
Aga2p: 4046-4252
ACP: 4274-4507
Myc: 4511-4540
Trp1: 5613-6287

GACGAAAGGGCCTCGTGATACGCCTATTTTTATAGGTTAATGTCATGATAATAATGGTTTCTTAGGACG
GATCGCTTGCCTGTAACTTACACGCGCCTCGTATCTTTTAATGATGGAATAATTTGGGAATTTACTCTG
TGTTTATTTATTTTTATGTTTTGTATTTGGATTTTAGAAAGTAAATAAAGAAGGTAGAAGAGTTACGGA
ATGAAGAAAAAAAAATAAACAAAGGTTTAAAAAATTTCAACAAAAAGCGTACTTTACATATATATTTA
TTAGACAAGAAAAGCAGATTAAATAGATATACATTCGATTAACGATAAGTAAAATGTAAAATCACAG
GATTTTCGTGTGTGGTCTTCTACACAGACAAGATGAAACAATTCGGCATTAATACCTGAGAGCAGGAA
GAGCAAGATAAAAGGTAGTATTTGTTGGCGATCCCCCTAGAGTCTTTTACATCTTCGGAAAACAAAAA
CTATTTTTTCTTTAATTTCTTTTTTTACTTTCTATTTTTAATTTATATATTTATATTAAAAAATTTAAATTA
TAATTATTTTTATAGCACGTGATGAAAAGGACCCAGGTGGCACTTTTCGGGGAAATGTGCGCGGAACC
CCTATTTGTTTATTTTTCTAAATACATTCAAATATGTATCCGCTCATGAGACAATAACCCTGATAAATG
CTTCAATAATATTGAAAAAGGAAGAGTATGAGTATTCAACATTTCCGTGTCGCCCTTATTCCCTTTTTT
GCGGCATTTTGCCTTCCTGTTTTTGCTCACCCAGAAACGCTGGTGAAAGTAAAAGATGCTGAAGATCA
GTTGGGTGCACGAGTGGGTTACATCGAACTGGATCTCAACAGCGGTAAGATCCTTGAGAGTTTTCGCC
CCGAAGAACGTTTTCCAATGATGAGCACTTTTAAAGTTCTGCTATGTGGCGCGGTATTATCCCGTATTG
ACGCCGGGCAAGAGCAACTCGGTCGCCGCATACACTATTCTCAGAATGACTTGGTTGAGTACTCACCA
GTCACAGAAAAGCATCTTACGGATGGCATGACAGTAAGAGAATTATGCAGTGCTGCCATAACCATGA
GTGATAACACTGCGGCCAACTTACTTCTGACAACGATCGGAGGACCGAAGGAGCTAACCGCTTTTTT
CACAACATGGGGGATCATGTAACTCGCCTTGATCGTTGGGAACCGGAGCTGAATGAAGCCATACCAAA
CGACGAGCGTGACACCACGATGCCTGTAGCAATGGCAACAACGTTGCGCAAACTATTAACTGGCGAA
CTACTTACTCTAGCTTCCCGGCAACAATTAATAGACTGGATGGAGGCGGATAAAGTTGCAGGACCACT
TCTGCGCTCGGCCCTTCCGGCTGGCTGGTTTATTGCTGATAAATCTGGAGCCGGTGAGCGTGGGTCTCG
CGGTATCATTGCAGCACTGGGGCCAGATGGTAAGCCCTCCCGTATCGTAGTTATCTACACGACGGGCA
GTCAGGCAACTATGGATGAACGAAATAGACAGATCGCTGAGATAGGTGCCTCACTGATTAAGCATTGG
TAACTGTCAGACCAAGTTTACTCATATATACTTTAGATTGATTTAAAACTTCATTTTTAATTTAAAAGG
ATCTAGGTGAAGATCCTTTTTGATAATCTCATGACCAAAATCCCTTAACGTGAGTTTTCGTTCCACTGA
GCGTCAGACCCCGTAGAAAAGATCAAAGGATCTTCTTGAGATCCTTTTTTTCTGCGCGTAATCTGCTGC
TTGCAAACAAAAAAACCACCGCTACCAGCGGTGGTTTGTTTGCCGGATCAAGAGCTACCAACTCTTTT
TCCGAAGGTAACTGGCTTCAGCAGAGCGCAGATACCAAATACTGTCCTTCTAGTGTAGCCGTAGTTAG
GCCACCACTTCAAGAACTCTGTAGCACCGCCTACATACCTCGCTCTGCTAATCCTGTTACCAGTGGCTG
CTGCCAGTGGCGATAAGTCGTGTCTTACCGGGTTGGACTCAAGACGATAGTTACCGGATAAGGCGCAG
CGGTCGGGCTGAACGGGGGGTTCGTGCACACAGCCCAGCTTGGAGCGAACGACCTACACCGAACTGA
GATACCTACAGCGTGAGCATTGAGAAAGCGCCACGCTTCCCGAAGGGAGAAAGGCGGACAGGTATCC
GGTAAGCGGCAGGGTCGGAACAGGAGAGCGCACGAGGGAGCTTCCAGGGGGGAACGCCTGGTATCTT
TATAGTCCTGTCGGGTTTCGCCACCTCTGACTTGAGCGTCGATTTTTGTGATGCTCGTCAGGGGGGCCG
AGCCTATGGAAAAACGCCAGCAACGCGGCCTTTTTACGGTTCCTGGCCTTTTGCTGGCCTTTTGCTCAC
ATGTTCTTTCCTGCGTTATCCCCTGATTCTGTGGATAACCGTATTACCGCCTTTGAGTGAGCTGATACCG
CTCGCCGCAGCCGAACGACCGAGCGCAGCGAGTCAGTGAGCGAGGAAGCGGAAGAGCGCCCAATACG
CAAACCGCCTCTCCCCGCGCGTTGGCCGATTCATTAATGCAGCTGGCACGACAGGTTTCCCGACTGGA
AAGCGGGCAGTGAGCGCAACGCAATTAATGTGAGTTACCTCACTCATTAGGCACCCCAGGCTTTACAC
TTTATGCTTCCGGCTCCTATGTTGTGTGGAATTGTGAGCGGATAACAATTTCACACAGGAAACAGCTAT
GACCATGATTACGCCAAGCTCGGAATTAACCCTCACTAAAGGGAACAAAAGCTGGGTACCCGACAGG
TTATCAGCAACAACACAGTCATATCCATTCTCAATTAGCTCTACCACAGTGTGTGAACCAATGTATCCA
GCACCACCTGTAACCAAAACAATTTTAGAAGTACTTTCACTTTGTAACTGAGCTGTCATTTATATTGAA

TTTTCAAAAATTCTTACTTTTTTTTTGGATGGACGCAAAGAAGTTTAATAATCATATTACATGGCATTA
CCACCATATACATATCCATATACATATCCATATCTAATCTTACTTATATGTTGTGGAAATGTAAAGAGC
CCCATTATCTTAGCCTAAAAAAACCTTCTCTTTGGAACTTTCAGTAATACGCTTAACTGCTCATTGCTAT
ATTGAAGTACGGATTAGAAGCCGCCGAGCGGGTGACAGCCCTCCGAAGGAAGACTCTCCTCCGTGCGT
CCTCGTCTTCACCGGTCGCGTTCCTGAAACGCAGATGTGCCTCGCGCCGCACTGCTCCGAACAATAAA
GATTCTACAATACTAGCTTTTATGGTTATGAAGAGGAAAAATTGGCAGTAACCTGGCCCCACAAACCT
TCAAATGAACGAATCAAATTAACAACCATAGGATGATAATGCGATTAGTTTTTTAGCCTTATTTCTGGG
GTAATTAATCAGCGAAGCGATGATTTTTGATCTATTAACAGATATATAAATGCAAAAACTGCATAACC
ACTTTAACTAATACTTTCAACATTTTCGGTTTGTATTACTTCTTATTCAAATGTAATAAAAGTATCAACA
AAAAATTGTTAATATACCTCTATACTTTAACGTCAAGGAGAAAAAACCCCGGATCGAATTCCCTACTT
CATACATTTTCAATTAAGATGAGATTTCCTTCAATTTTTACTGCAGTTGTCTTCGCAGCATCCTCCGCAT
TAGCTGCTCCAGCAAACACTACAGCTGAAGATGAAACGGCACAAATTCCAGCTGAAGCTGTCATCGGT
TACTTAGGTTTAGAAGGGGATAGCGATGTTGCTGCCTTGCCATTATCCGATAGCACAAATAACGGGTC
ATTGTCCACAAATACTACTATTGCCAGCATTGCTGCTAAAGAAGAAGGGGTACAATTAGATAAAAGAG
AGGCCGAAGCTTAGGCGGCTAATGTCGACATAGGTCAAGGATCCGGAGGTGGCTCTGGTGGAGGGGG
ATCAGGAGGTGGTGGCTCCGGTGGAGGTGGTTCGGGCGGAGGTGGTTCCGGTGGAGGAGGTTCTGGT
GGAGGCGGTAGCCAGGAACTGACAACTATATGCGAGCAAATCCCCTCACCAACTTTAGAATCGACACC
ATACTCTTTGTCAACGACTACTATTTTGGCCAACGGGAAGGCAATGCAAGGAGTTTTTGAATATTACA
AATCAGTAACGTTTGTCAGTAATTGCGGTTCTCACCCCTCAACAACTAGCAAAGGCAGCCCCATAAAC
ACACAGTATGTTTTTAAGGACAATAGCTCGACtagtATGAGCACTATCGAAGAACGTGTTAAGAAAATTA
TCGGCGAACAGCTGGGCGTTAAGCAGGAAGAAGTTACCAACAATGCTTCTTTCGTTGAAGACCTGGGC
GCCGATTCTCTTGACACCGTTGAGCTGGTAATGGCTCTGGAAGAAGAGTTTGATACTGAGATTCCAGA
CGAAGAAGCTGAGAAAATCACCACCGTTCAGGCTGCCATTGATTACATCAACGGCCACCAGGCTAGCG
AACAAAAGTTAATTTCTGAAGAGGACTTGTAATAGCATGCAGATCTGATAACAACAGTGTAGATGTAA
CAAAATCGACTTTGTTCCCACTGTACTTTTAGCTCGTACAAAATACAATATACTTTTCATTTCTCCGTAA
ACAACATGTTTTCCCATGTAATATCCTTTTCTATTTTTCGTTCCGTTACCAACTTTACACATACTTTATAT
AGCTATTCACTTCTATACACTAAAAAACTAAGACAATTTTAATTTTGCTGCCTGCCATATTTCAATTTGT
TATAAATTCCTATAATTTATCCTATTAGTAGCTAAAAAAAGATGAATGTGAATCGAATCCTAAGAGAA
TTGAGCTCCAATTCGCCCTATAGTGAGTCGTATTACAATTCACTGGCCGTCGTTTTACAACGTCGTGAC
TGGGAAAACCCTGGCGTTACCCAACTTAATCGCCTTGCAGCACATCCCCCTTTCGCCAGCTGGCGTAAT
AGCGAAGAGGCCCGCACCGATCGCCCTTCCCAACAGTTGCGCAGCCTGAATGGCGAATGGCGCGACG
CGCCCTGTAGCGGCGCATTAAGCGCGGCGGGTGTGGTGGTTACGCGCAGCGTGACCGCTACACTTGCC
AGCGCCCTAGCGCCCGCTCCTTTCGCTTTCTTCCCTTCCTTTCTCGCCACGTTCGCCGGCTTTCCCCGTC
AAGCTCTAAATCGGGGGCTCCCTTTAGGGTTCCGATTTAGTGCTTTACGGCACCTCGACCCCAAAAAA
CTTGATTAGGGTGATGGTTCACGTAGTGGGCCATCGCCCTGATAGACGGTTTTTCGCCCTTTGACGTTG
GAGTCCACGTTCTTTAATAGTGGACTCTTGTTCCAAACTGGAACAACACTCAACCCTATCTCGGTCTAT
TCTTTTGATTTATAAGGGATTTTGCCGATTTCGGCCTATTGGTTAAAAAATGAGCTGATTTAACAAAAA
TTTAACGCGAATTTTAACAAAATATTAACGTTTACAATTTCCTGATGCGGTATTTTCTCCTTACGCATCT
GTGCGGTATTTCACACCGCAGGCAAGTGCACAAACAATACTTAAATAAATACTACTCAGTAATAACCT
ATTTCTTAGCATTTTTGACGAAATTTGCTATTTTGTTAGAGTCTTTTACACCATTTGTCTCCACACCTCC
GCTTACATCAACACCAATAACGCCATTTAATCTAAGCGCATCACCAACATTTTCTGGCGTCAGTCCACC
AGCTAACATAAAATGTAAACTTTCGGGGCTCTCTTGCCTTCCAACCCAGTCAGAAATCGAGTTCCAATC
CAAAAGTTCACCTGTCCCACCTGCTTCTGAATCAAACAAGGGAATAAACGAATGAGGTTTCTGTGAAG
CTGCACTGAGTAGTATGTTGCAGTCTTTTGGAAATACGAGTCTTTTAATAACTGGCAAACCGAGGAAC
TCTTGGTATTCTTGCCACGACTCATCTCCATGCAGTTGGACGATATCAATGCCGTAATCATTGACCAGA
GCCAAAACATCCTCCTTAGGTTGATTACGAAACACGCCAACCAAGTATTTCGGAGTGCCTGAACTATT
TTTATATGCTTTTACAAGACTTGAAATTTTCCTTGCAATAACCGGGTCAATTGTTCTCTTTCTATTGGGC
ACACATATAATACCCAGCAAGTCAGCATCGGAATCTAGAGCACATTCTGCGGCCTCTGTGCTCTGCAA
GCCGCAAACTTTCACCAATGGACCAGAACTACCTGTGAAATTAATAACAGACATACTCCAAGCTGCCT
TTGTGTGCTTAATCACGTATACTCACGTGCTCAATAGTCACCAATGCCCTCCCTCTTGGCCCTCTCCTTT
TCTTTTTTCGACCGAATTAATTCTTAATCGGCAAAAAAAGAAAAGCTCCGGATCAAGATTGTACGTAA
GGTGACAAGCTATTTTTCAATAAAGAATATCTTCCACTACTGCCATCTGGCGTCATAACTGCAAAGTAC
ACATATATTACGATGCTGTCTATTAAATGCTTCCTATATTATATATATAGTAATGTCGTTTATGGTGCAC
TCTCAGTACAATCTGCTCTGATGCCGCATAGTTAAGCCAGCCCCGACACCCGCCAACACCCGCTGACG
CGCCCTGACGGGCTTGTCTGCTCCCGGCATCCGCTTACAGACAAGCTGTGACCGTCTCCGGGAGCTGC
ATGTGTCAGAGGTTTTCACCGTCATCACCGAAACGCGCGA
